# Comparison of the 24 h Dietary Recall of Two Consecutive Days, Two Non-Consecutive Days, Three Consecutive Days, and Three Non-Consecutive Days for Estimating Dietary Intake of Chinese Adult

**DOI:** 10.3390/nu14091960

**Published:** 2022-05-07

**Authors:** Kun Huang, Liyun Zhao, Qiya Guo, Dongmei Yu, Yuxiang Yang, Qiuye Cao, Xiaolin Yuan, Lahong Ju, Shujuan Li, Xue Cheng, Xiaoli Xu, Hongyun Fang

**Affiliations:** NHC Key Laboratory of Trace Element Nutrition, National Institute for Nutrition and Health, Chinese Center for Disease Control and Prevention, Beijing 100050, China; 15550807252@163.com (K.H.); zhaoly@ninh.chinacdc.cn (L.Z.); guoqy@ninh.chinacdc.cn (Q.G.); yu_dongmei@126.com (D.Y.); yxyang_ninhccdc@126.com (Y.Y.); caoqy@ninh.chinacdc.cn (Q.C.); yuanxl@ninh.chinacdc.cn (X.Y.); julh@ninh.chinacdc.cn (L.J.); lisj@ninh.chinacdc.cn (S.L.); chengxue@ninh.chinacdc.cn (X.C.); xuxl@ninh.chinacdc.cn (X.X.)

**Keywords:** 24 h dietary recall, consecutive, accuracy, dietary intake, nutrition survey

## Abstract

The specific forms of 24 h dietary recall used by national nutrition surveys differ, such as two non-consecutive days and three consecutive days. However, it is unclear which form of 24 h dietary recall is more accurate in the Chinese population. The purpose of this study was to compare the performance of 24 h recalls on two consecutive days (C2), three consecutive days (C3), two non-consecutive days (NC2), and three non-consecutive days (NC3) in estimating Chinese adult dietary intake. A total of 595 participants completed more than twenty-three 24 h recalls. The average of all completed 24 h recalls of each subject was defined as the individual’s true dietary intake. The dietary intake in the four scenarios of 24 h recalls was calculated using the within-person mean (WPM) method and National Cancer Institute (NCI) method and compared with the true values. Equivalent testing was used to evaluate whether scenarios NC2 and C3 were equivalent. Bias and mean bias were used as a measure of precision and accuracy, respectively. For the WPM method, the precision between the four scenarios was similar. For mean, the accuracy between the four scenarios was similar, yielding estimates that were close to the true intakes. However, for percentiles, the accuracy in descending order was scenario NC3, C3, NC2, and C2. Furthermore, the difference between two and three days was greater than that between consecutive and non-consecutive days. In most case, the distribution of dietary intakes calculated from scenarios NC2 and C3 was equivalent with equivalence margins of 5% (*p* < 0.05). Usually, the NCI method was significantly more accurate than the WPM method. We concluded that three non-consecutive 24 h recalls relative to three consecutive days increases accuracy. Two non-consecutive days can be substituted to some extent for three consecutive days. The new form of 24 h recall needs to be used with caution when applied practically in the China nutrition surveys. Furthermore, using the NCI method to calculate dietary intake from 24 h recall may be a way to reduce costs and increase accuracy.

## 1. Introduction

Diet has been associated with many important health outcomes, such as cancer, cardiovascular diseases, and diabetes [1,2]. Incorrect estimates of dietary status may lead to incorrect risk estimates of chronic diseases. However, a dietary survey is time-consuming and requires a lot of human and material resources. How to use a cost-saving instrument to accurately estimate dietary status remains a global public health challenge. At present, a variety of dietary survey methods exists, including dietary records, weighing method, chemical analysis, 24 h dietary recall, and food frequency questionnaires (FFQ) [3,4,5]. Of the many methods available, the 24 h recall is widely used in national nutrition surveys because of its validity, high precision, and elevated response rate [6,7,8]. This method is a subjective recall method that requires a direct face-to-face or telephone interview, and data can also be collected in the form of online review self-reports via the Internet. The method consists of accurately recalling, describing, and quantifying the intake of all foods and beverages consumed in the past 24 h prior to the interview [9]. Enough repeated 24 h recalls can reduce within-individual variation and more accurately estimate usual dietary intake [10]. However, the number of survey-days needed to obtain dietary data with sufficient statistical precision at the population level ranged from 3 days for energy to 41 days for vitamin A, and for the individual level even more days were needed [11]. Although fewer survey days are needed within larger groups, as the number of 24 h recalls increases, especially in large population-based studies, the human and material resources required increase substantially [11,12].

National nutrition surveys are essential for assessing population-level dietary intake and developing public health policies to promote nutrition and health. The 24 h dietary recall method used for national-level nutrition surveys varies considerably among countries. The national nutrition surveys conducted in the United States, Canada, Australia, and Mexico all use two non-consecutive 24 h recalls [6,7,13,14]. In Asia, the Korea National Health and Nutrition Examination Survey (KNHANES) uses single 24 h recall [15], and China Nutrition and Health Surveillance (CNHS) uses three consecutive days including two weekdays and one weekend [16]. There is no existing standard to judge which approach is the best. In addition, some studies have shown that food consumption on consecutive days may be correlated [17,18,19]. For example, a high consumption on one day may be followed by a very low consumption on the next day. Currently, there are relatively few studies on the use of new forms of 24 h recalls in Chinese populations.

In this study, participants completed a total of twenty-eight 24 h recalls in a single year. In addition, the average of over twenty-three 24 h recalls was regarded as the gold standard. We compared the differences in estimating the population-level dietary intake of Chinese adults between the four scenarios: two consecutive days, two non-consecutive days, three non-consecutive days, and three consecutive days. Furthermore, the accuracy of estimates calculated by the within-person mean (WPM) method and the National Cancer Institute (NCI) method was compared for the above four scenarios. We aimed to explore whether the cost and participants’ burden of implementing national nutrition surveillance could be decreased by reducing the number of 24 h recalls or collecting non-consecutive days based on the existing accuracy. Furthermore, these findings can provide further evidence for the use of new forms of 24 h recalls in conducting nutrition surveys in China.

## 2. Materials and Methods

### 2.1. Study Design and Participants

To investigate the impact of the number of 24 h recalls on dietary intake assessments in southern and northern Chinese populations, Zhejiang Province and Shanxi Province were selected as representative provinces. One urban and one rural survey site were selected from each province. Ninety-nine male and ninety-nine female subjects were recruited from each survey site. The selected survey sites were required to have enough experienced investigators who have participated in the CNHS. Purposive sampling was used to recruit participants who were cooperative and could be surveyed repeatedly. The exclusion criteria for participants were as follows: age over 60 years or under 18 years; disabled or mobility impaired; communication impairment; patients with severe hypertension, hyperglycemia, or hyperlipidemia. The survey was conducted quarterly for seven consecutive days from December 2019 to December 2020. Finally, 780 eligible participants from four survey sites completed twenty-eight 24 h recalls.

In this study, after some survey days were cleared for reporting incredible energy intake (outside the range of 600 to 4200 kcal per day for male or 400 to 3500 kcal for female), 28 subjects were excluded for less than twenty-three 24 h recalls [20,21]. When enough repeated 24 h recalls can represent an individual’s true dietary intake, the dietary intake estimates should stabilize as the number of 24 h recalls increases. Therefore, for those subjects who completed twenty-eight 24 h recalls, subjects were included in this analysis if the difference of the average energy intake calculated from 23 to 27 (28 minus 1 to 28 minus 5) days was less than 5% compared to 28 days. For the other subjects, similar calculations were performed to determine whether the subjects were included. Ultimately, 595 eligible participants were included in the analysis.

The study protocol was approved by the Ethics Committee of the Chinese Center for Disease Control and Prevention (No. 201519-B), and all participants signed an informed consent form before participating.

### 2.2. Data Collection and Measurements

A standard set of questionnaires was designed to collect information from subjects, including basic information, health status, dietary information, and condiments consumption.

The questionnaire’s information was collected by the investigators using in person interviews in households. In the repeated survey, participants were followed up with by the same investigator. Investigators must receive uniform training from the national working group and pass an examination before they can conduct on-site surveys.

### 2.3. Dietary Intake Assessment

The 24 h dietary recall method was used to collect dietary information from participants who were asked to recall their food consumption in the past 24 h, including staple foods, side dishes, snacks, fruits, and beverages. Daily energy and nutrient intakes of the participants were calculated based on the Chinese Food Composition Tables [22]. To comprehensively compare the estimated dietary intakes under the four scenarios, we included 25 dietary components that were frequently assessed, including energy, 19 nutrients (fat, carbohydrate, protein, dietary fiber, cholesterol, vitamin A, vitamin C, vitamin E, vitamin B1, vitamin B2, vitamin B3, vitamin B9, calcium, iron, zinc, magnesium, sodium, potassium, phosphorus) and 5 foods (wheat, pork, vegetables, milk, beans).

### 2.4. Data Sets

Scenario C2 was defined as two consecutive 24 h recalls. Scenario C3 was defined as three consecutive 24 h recalls. Scenario NC2 was defined as two non-consecutive 24 h recalls within a week, i.e., the first and second days were separated by a minimum of one day and a maximum of five days. Scenario NC3 was defined as three non-consecutive 24 h recalls within a week, i.e., two adjacent days were separated by a minimum of one day and the first and third days were separated by a maximum of five days. Seasonal and weekend effects may lead to different results at different survey times. For example, the estimates for Monday and Tuesday in summer are different from the estimates for Friday and Saturday in winter. To overcome this limitation, we generated data sets containing all possible combinations of collection days, e.g., for scenario NC3, a data set of Monday, Wednesday, Friday; then Monday, Wednesday, Saturday; then Monday, Wednesday, Sunday; and so on, until all combinations were drawn. It should be noted that the maximum interval between two days is five days, because in practice, 24 h recalls are completed during a week. Thus, scenarios C2, C3, NC2, and NC3 generated 24, 20, 60, and 40 data sets, respectively.

### 2.5. Statistical Analysis

We calculated dietary intake estimates for the four scenarios using the NCI method and WPM method, respectively. In addition, the NCI model was adjusted for age, sex, and weekend effects’ covariates [23,24]. The true dietary intake was defined as the average of all 24 h recalls (twenty-three or more) which was considered as the ‘gold standard’ for dietary intake [20].

To compare estimates from different scenarios and methods, we calculated bias B for each dietary component,

(1) $$B_{i}=E_{i}-T_{i}$$

mean bias (MB),

(2) $$MB_{i}=\frac{\sum_{i=1}^{N}\left(E_{i}-T_{i}\right)}{N}$$

mean relative bias (MRB),

(3) $$MRB_{i}=\left|\frac{MB_{i}}{T_{i}}\right|\times 100\%$$

and mean squared error (MSE),

(4) $$MSE_{i}=\frac{\sum_{i=1}^{N}{\left(E_{i}-T_{i}\right)}^{2}}{N}$$

where $E_{i}$ and $T_{i}$ are defined as the estimated and true value of the parameter for the dietary components i, respectively, and N is the number of data sets for each scenario.

We used equivalence testing with equivalence margins of 1, 5, and 10% of the estimates (for means and percentiles) from scenario C3 to evaluate whether the parameters from scenario NC2 and C3 were equivalent [25]. Ninety percent confidence intervals with a confidence level α equal to 0.05 were calculated as the equivalence test relative to the two one-sided tests [26].

The NCI method and other analyses were conducted by SAS version 9.4 (SAS Institute Inc., Cary, NC, USA), and all plots were constructed by R version 4.1.2.

## 3. Results

### 3.1. Subjects’ Characteristics

The study population consisted of 595 participants, including 48.9% aged between 18 and 40, 52.1% female, 51.1% urban population, and 54.5% from the northern region. Most participants (86.4%) completed 28 qualified 24 h recalls. The characteristic distribution of the subjects from the south and north was similar. The detailed characteristics of the study population are presented in Table 1.

### 3.2. Comparison of Four Scenarios Based on WPM Method

**Figure 1:**
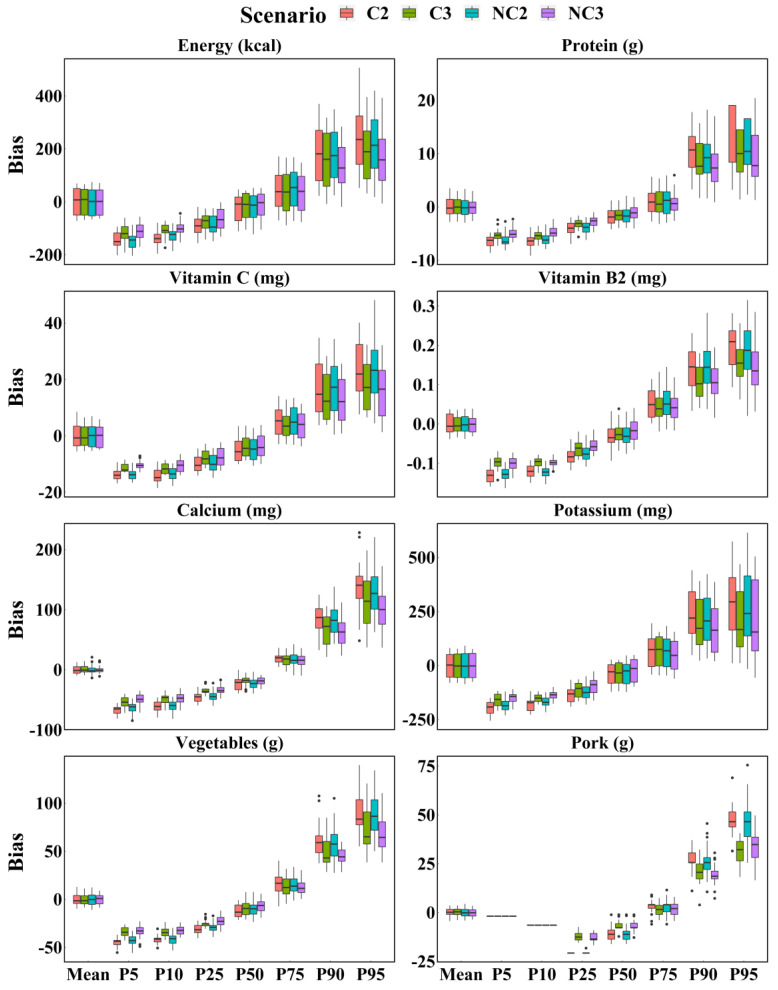
Boxplot of biases of intake calculated for energy, protein, vitamin C, vitamin B2, calcium, potassium, vegetables, and pork based on each scenario with WPM method. C2 = Two consecutive 24 h recalls; C3 = Three consecutive 24 h recalls; NC2 = Two non-consecutive 24 h recalls; NC3 = Three non-consecutive 24 h recalls.

Figure 1 shows boxplots of the biases in each scenario estimated by the WPM method, confirming similar precision between the scenarios. The differences in the spread of bias between the four scenarios were few, especially when compared to mean; however, those had changed with dietary components and parameters. Regardless of the dietary components, there was a tendency of decreasing precision from the 5th to the 95th percentile for each scenario. Interestingly, except for mean and median, three 24 h recalls were more accurate than two 24 h recalls whether the survey days were consecutive or not. Greater differences were observed in foods between three 24 h recalls and two 24 h recalls, such as vegetables and pork.

**Figure 2:**
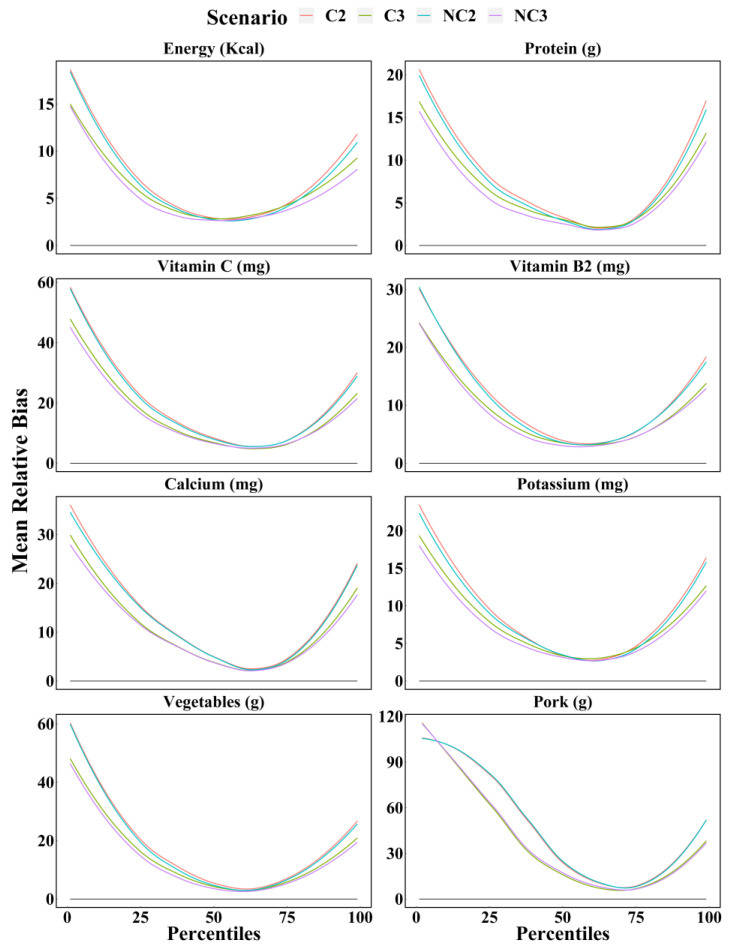
Mean relative bias of the percentiles (from 1st to 99th) of intake calculated for energy, protein, vitamin C, thiamin, calcium, potassium, vegetables, and pork based on each scenario with WPM method. C2 = Two consecutive 24 h recalls; C3 = Three consecutive 24 h recalls; NC2 = Two non-consecutive 24 h recalls; NC3 = Three non-consecutive 24 h recalls.

Figure 2 shows the mean relative biases of four scenarios estimated by the WPM method for dietary components. The accuracy between scenario NC3 and C3 are similar, as are scenario NC2 and C2. It is interesting to note that, for any food and nutrient, the percentile estimates (from 1st to 99th) of dietary intake calculated using three 24 h recalls were closer to the true values than those calculated using two 24 h recalls. In most cases, the mean relative biases of dietary intake calculated for these four scenarios were from largest to smallest for scenario C2, NC2, C3, and NC3, but the differences between the same number of days were small. As expected, the number of 24 h recalls was the main factor affecting the accuracy of dietary intake estimates, rather than whether multiple 24 h recalls were consecutive. This effect was more pronounced for certain dietary components, such as fat, sodium, and milk. Furthermore, the greater differences between two and three 24 h recalls were observed at both ends of the percentiles.

Table 2 shows the true value, mean bias, mean relative bias, and MSE for the mean and some percentiles of the dietary intake distribution based on the WPM method in each evaluated scenario. The mean relative bias and MSE of the presenting percentiles were similar in scenarios NC3 and C3 and were much smaller than those in scenarios NC2 and C2. The scenarios NC3 and C3 yielded more accurate estimates for the percentiles than scenarios NC2 and C2, particularly for the 5th and 95th percentiles. With few exceptions, the accuracy of scenario NC3 was the highest while scenario C2 was the lowest. The performances of estimating the mean between compared scenarios were close, yielding estimates close to the true values. Over all scenarios and dietary components, the range of the mean relative bias in the mean of the dietary intake varied from 0.00% to 1.68%. However, the corresponding ranges of the 5th and 95th percentiles were much wider, especially for the foods with low consumption frequency, such as pork, where the corresponding ranges were from 100% to 100% and from 27.07% to 39.92%, respectively.

### 3.3. Equivalence Testing between Scenario C3 and NC2

Table 3 shows that, in most cases, the estimates via scenarios C3 and NC2 are functionally identical for applied use. The equivalence testing was statistically significant by (*p* < 0.05) when equivalence testing was with equivalence margins of 10% of scenario C3 estimates. For the means, scenarios C3 and NC2 were equivalent within 5% error for all dietary components, and they were equivalent even within 1% error for most nutrients, such as energy, protein, vitamin B1, vitamin B2, and zinc. However, equivalence margins of percentiles with statistical significance were larger than those of the mean. For example, the equivalence margins for fat, vitamin C, sodium, and vegetables at the 5th and 10th percentiles were mostly in the range of 5 to 10%. In addition, the 90% confidence interval for foods with low consumption frequencies (such as pork and milk) and for nutrients with wide variations in different foods (such as vitamin A and sodium), was wider than for other dietary components.

### 3.4. Comparison of Four Scenarios between WPM and NCI Method

**Figure 3:**
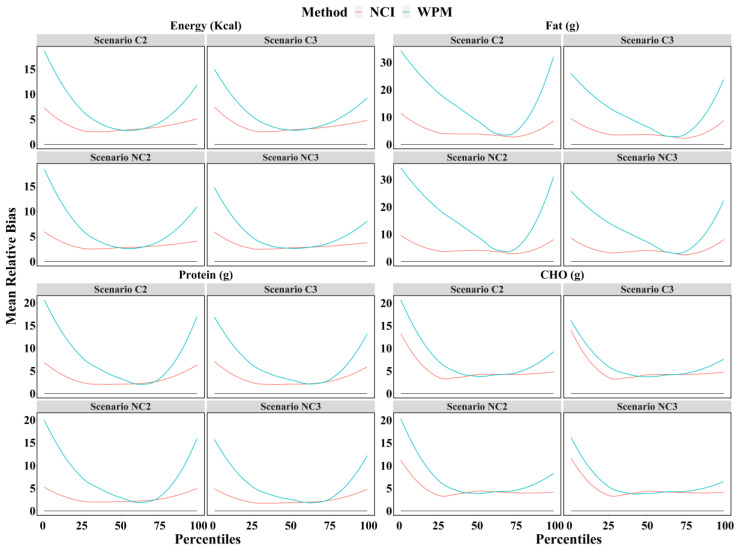
The mean relative biases of the percentiles (from 1st to 99th) of intake calculated for energy, protein, fat, and CHO based on each scenario with the WPM and NCI method. NCI = National Cancer Institute; WPM = Within-person mean; C2 = Two consecutive 24 h recalls; C3 = Three consecutive 24 h recalls; NC2 = Two non-consecutive 24 h recalls; NC3 = Three non-consecutive 24 h recalls; CHO = Carbohydrate.

Figure 3 shows that the mean relative biases for the percentiles (from 1st to 99th) of dietary intake estimated by the NCI method were significantly less than those estimated by the WPM method, especially for the percentiles outside the interquartile range. For example, the mean relative bias for the 25th percentile of energy intake by the WPM method was twice as high as the result estimated by the NCI method. These results illustrated that the NCI method always provides more accurate estimates than the WPM method, regardless of the number of 24 h recalls and whether the survey days were consecutive or not.

More results are presented in the supplementary material (Table S1, Figures S1–S3), including: the boxplot of bias for each scenario and each dietary component with the WPM method; the smooth line of mean relative bias of the percentiles (from 1st to 99th) for all dietary components based on each scenario with the WPM method; the mean bias, mean relative bias, and MSE of estimates in the 5th, 10th, 25th, 50th, 75th, 90th, and 95th percentiles as well as the mean for each scenario and dietary components with the WPM method; as well as the smooth line of mean relative bias of the percentiles (from 1st to 99th) for all dietary components based on each scenario with the NCI and WPM methods.

## 4. Discussion

The China Nutrition and Health Survey (CNHS) is a national nutrition survey to understand the dietary structure, nutrition, and health status of the population and its changing tendencies [27]. The findings of CNHS can reveal the impact of socio-economic factors on the nutrition and health status among the Chinese population and provide science-based evidence for making and conducting public health policies. The reliability of the CNHS dietary survey methodology is critical as an important basis for assessing population nutritional status. According to previous monitoring reports, the average number of participants in each dietary survey reached about 80,000, which would bring a very heavy workload [27].

The primary aims of this study were to find the new form of 24 h recall to reduce the cost invested and the burden on subjects in CNHS. Hence, we compared four scenarios for estimating dietary intake using the 24 h recall. The results showed that, with a few dietary exceptions, the three non-consecutive 24 h recalls outperformed compared with the other three scenarios. The bias distribution was similar between the four scenarios, especially at the mean and median, indicating that the four scenarios have the same precision. In other words, for each scenario, the stability was similar when using samples drawn from different times of the year to estimate dietary intake. Additionally, the range of bias tended to increase from the 5th to 95th percentile, indicating that the intake fluctuated more across time for the high intake group.

Previous studies have shown that food intake is significantly reduced from winter to summer, with the highest intake of energy, protein, and fat occurring in winter and the lowest in summer [28,29,30]. Moreover, the intake of energy and carbohydrates is more on weekends than on weekdays [31]. Both seasonal and weekend effects can affect dietary intake [28,29,30,31]. To overcome this limitation on comparing the accuracy of the four scenarios, we calculated the mean of all samples for each scenario as a representative value to compare the accuracy between the four scenarios. The results showed that from the 1st to 99th percentile, three non-consecutive days appeared to be the most accurate scenario, followed by three consecutive days. The accuracy between two non-consecutive days and two consecutive days was close, but the former was better. The above results indicated that for the dietary intake calculated by the WPM method, the more non-consecutive 24 h recalls were collected, the more accurate the data were. Additionally, the number of 24 h recalls had a greater impact on the accuracy than whether it was consecutive or not. This may be because the main factor affecting the group dietary intake is the within-person variation which decreases with the increase in the number of 24 h recalls [32]. In addition, there is an association between consecutive days, for example, one day of high intake followed by the next day of low intake, so non-consecutive days are recommended to collect dietary information [17,18,19,33].

These results are consistent with studies in African American youth, which found that the reliability estimates of energy, fat, fruit, and vegetable intake increased with the number of 24 h recalls [20]. Similar results have also been reported by Ma et al. in middle-aged white women, which indicated that estimates of energy intake from the two recalls better approximated true energy expenditure than did the first recall, and the three recalls further improved the estimate [34]. Moreover, one study used 16 food records collected over a year as a reference to compare three consecutive-day and three random-day records of dietary intake, and found that for energy, protein, fat, and calcium, the random days were more accurate than the consecutive day, which is consistent with our findings [17].

Subsequently, we compared the means and percentiles of dietary intake estimated for the four scenarios. The results showed that the means estimated for all four scenarios were very close to the true values, suggesting that accurate results were obtained for the average dietary intake, regardless of two or three days, consecutive or non-consecutive days. For percentiles, however, the mean relative bias and MSE of three non-consecutive days were minimal, indicating that it is not only a more accurate scenario than the others, but also less affected by extreme intakes. Although we believe that three non-consecutive 24 h recalls have a higher accuracy, it does not reduce participants’ burden and cost, so it is not suitable for large-scale surveys such as CNHS. Therefore, we explored the accuracy lost by using two non-consecutive days instead of three consecutive days. The results found that for the mean and median, the two scenarios were equivalent within 5% error; however, for the 5th and 10th percentiles, the error expanded to 10%. The above results hold for most dietary components, especially energy and macronutrients. However, they may not be suitable to nutrients with high variability in food content and foods with low frequency, such as vitamin A, sodium, and pork. Furthermore, for these dietary components, the parameters estimated for each scenario differed significantly from the true values. Some statistical methods may be able to address these issues, for instance, the NCI method uses a short-term 24 h dietary recalls to estimate usual dietary intake [21].

Another aim of this study was to compare the accuracy of the four scenarios by different methods. A previous study has shown that dietary intakes estimated by the NCI method using three consecutive 24 h recalls were closer to the true values than the WPM method at both the group and individual levels [35]. As with the results of previous studies, these results found that for each scenario and each dietary component, the NCI method performed better. The NCI method can obtain more accurate estimates by eliminating within-person variation and shrinking the intake distribution toward the mean [36]. However, since the intake estimates for the NCI method were not compared across the four scenarios, it could not be determined which scenario was more accurate. In a follow-up study, we will compare the differences between the four scenarios under the NCI method to find the most accurate and least costly scenario for the 24 h dietary recall with statistical correction via the NCI method.

A limitation of the present study is that two or three 24 h recalls were drawn from multiple replicate 24 h recalls, so they may not capture the effect of reducing the number of replicate 24 h recalls on the participants. However, we speculate that using only two non-consecutive 24 h recalls in the population would have yielded more accurate results than the present study. In addition, this study only explored Chinese adults from 18 to 60 years, excluding minors and the elderly, so caution is needed when extrapolating the results.

These findings provided support for the adoption of a new form of 24 h recall in the CNHS. Firstly, this study provided the average of twenty-eight 24 h recalls as true values, which were obtained from actual surveys rather than simulations. Second, we compared differences between four scenarios of 24 h recall among energy, nutrients, and foods to illustrate the generalizability of the results, which is consistent with the purpose of the CNHS dietary survey. Further, we compared all possible survey days drawn from the week distributed over the four seasons, because each monitoring site conducts dietary surveys at different times in the actual survey.

## 5. Conclusions

In the Chinese adult population, the three non-consecutive 24 h recalls provide a more accurate estimate of dietary intake, but a little improvement relative to three consecutive days. For most foods and nutrients, two non-consecutive days can replace three consecutive days, but can impair some accuracy. For all four scenarios of 24 h recalls, the NCI method achieved significantly more accurate results than the WPM method. Hence, in the China nutrition surveys, we recommend that two non-consecutive 24 h dietary recall is used to collect dietary data and the NCI method is used to correct within-person variation.

## Figures and Tables

**Table 1:** The characteristics of participants aged from 18 to 60.

Characteristics	Total	Northern	Southern
N	595 (100%)	324 (54.5%)	271 (45.5%)
Age group (year)			
18–39	291 (48.9%)	170 (52.5%)	121 (44.6%)
40–60	304 (51.1%)	154 (47.5%)	150 (55.4%)
Gender			
Male	285 (47.9%)	154 (47.5%)	131 (48.3%)
Female	310 (52.1%)	170 (52.5%)	140 (51.7%)
Urban or Rural			
Urban	304 (51.1%)	164 (50.6%)	140 (51.7%)
Rural	291 (48.9%)	160 (49.4%)	131 (48.3%)
Education level			
Primary school or below	34 (5.7%)	11 (3.4%)	23 (8.5%)
Middle school	186 (31.3%)	130 (40.1%)	56 (20.7%)
High school and above	375 (63%)	183 (56.5%)	192 (70.8%)
Household income level *			
Low	9 (1.5%)	8 (2.5%)	1 (0.4%)
Medium	124 (20.8%)	110 (34.0%)	14 (5.2%)
High	204 (34.3%)	56 (17.2%)	148 (54.5%)
Unclear	258 (43.4%)	150 (46.3%)	108 (39.9%)
BMI (Kg/m^2^)			
18.5<	13 (2.2%)	4 (1.2%)	9 (3.3%)
18.5–23.9	300 (50.4%)	143 (44.1%)	157 (57.9%)
≥24	282 (47.4%)	177 (54.7%)	105 (38.8%)
The number of 24 h recalls			
23	4 (0.7%)	1 (0.3%)	3 (1.1%)
24	4 (0.7%)	4 (1.2%)	0 (0%)
25	5 (0.8%)	1 (0.3%)	4 (1.5%)
26	12 (2.0%)	5 (1.5%)	7 (2.6%)
27	56 (9.4%)	29 (9.0%)	27 (10.0%)
28	514 (86.4%)	284 (87.7%)	230 (84.8%)

*—Household Income Level: low (<20,000 RMB), medium (20,000–50,000 RMB), high (≥50,000 RMB), and Unclear (unknown or refused to answer); BMI—Body Mass Index.

**Table 2:** Mean bias, mean relative bias, and MSE of estimates obtained with each scenario for each dietary component based on the WPM method.

Dietary Components	Parameter	True Value	Mean Bias (Mean Relative Bias %)				MSE			
			C2	NC2	C3	NC3	C2	NC2	C3	NC3
Protein (g)	Mean	66.47	−0.01 (−0.01)	0.02 (0.03)	−0.04 (−0.06)	0.03 (0.05)	3.34	2.97	3.16	2.77
	P05	38.46	−6.48 (16.84)	−6.31 (16.40)	−5.29 (13.75)	−4.93 (12.82)	43.03	40.86	29.42	25.33
	P10	43.48	−6.42 (14.76)	−6.04 (13.88)	−5.36 (12.33)	−4.64 (10.67)	42.73	37.34	29.73	22.72
	P25	51.70	−3.98 (7.69)	−3.84 (7.42)	−3.11 (6.01)	−2.72 (5.27)	17.24	15.82	10.54	8.30
	P50	63.66	−1.78 (3.18)	−1.48 (2.78)	−1.53 (2.75)	−0.93 (2.34)	5.56	4.33	4.28	3.01
	P75	79.31	1.24 (2.68)	1.06 (2.68)	0.98 (2.56)	0.69 (2.14)	7.15	6.49	6.44	4.58
	P90	91.21	10.33 (11.32)	9.48 (10.39)	8.46 (9.28)	7.71 (8.45)	124.67	107.60	87.89	75.06
	P95	103.04	13.92 (13.51)	11.79 (11.44)	10.69 (10.38)	9.26 (8.99)	237.60	175.03	147.45	113.17
Zinc (mg)	Mean	9.90	−0.01 (−0.07)	0 (0.04)	−0.01 (−0.14)	0.01 (0.07)	0.12	0.11	0.11	0.10
	P05	5.63	−1.08 (19.13)	−1.03 (18.30)	−0.91 (16.19)	−0.85 (15.08)	1.18	1.09	0.87	0.76
	P10	6.17	−0.87 (14.11)	−0.8 (12.89)	−0.72 (11.63)	−0.58 (9.33)	0.79	0.67	0.54	0.39
	P25	7.53	−0.63 (8.42)	−0.58 (7.71)	−0.50 (6.66)	−0.42 (5.63)	0.44	0.40	0.29	0.24
	P50	9.31	−0.19 (3.36)	−0.19 (2.87)	−0.12 (3.15)	−0.09 (2.62)	0.13	0.11	0.13	0.09
	P75	11.82	0.04 (2.98)	0.08 (3.06)	0.02 (2.93)	0.03 (2.82)	0.17	0.19	0.17	0.17
	P90	14.06	1.16 (8.28)	1.07 (7.61)	0.98 (6.99)	0.87 (6.21)	1.86	1.54	1.36	1.08
	P95	16.06	1.75 (10.89)	1.52 (9.48)	1.28 (8.1)	1.13 (7.09)	3.90	3.03	2.40	1.87
Vitamin C (mg)	Mean	65.68	−0.06 (−0.09)	0.01 (0.01)	−0.21 (−0.31)	−0.03 (−0.04)	15.88	16.13	14.30	15.65
	P05	28.59	−13.71 (47.96)	−13.71 (47.97)	−11.32 (39.59)	−10.4 (36.37)	191.38	191.45	130.65	110.08
	P10	35.41	−14.13 (39.91)	−13.44 (37.94)	−11.51 (32.49)	−10.53 (29.75)	205.34	184.91	135.68	115.61
	P25	45.32	−9.88 (21.81)	−9.52 (21.00)	−7.72 (17.03)	−7.39 (16.31)	105.04	99.41	66.08	65.57
	P50	61.68	−4.69 (8.59)	−4.75 (8.07)	−3.92 (7.31)	−3.60 (6.81)	38.72	36.95	29.25	27.16
	P75	80.23	4.98 (6.95)	5.01 (6.84)	3.85 (5.77)	3.79 (5.98)	50.78	50.25	36.61	35.68
	P90	101.46	16.76 (16.52)	16.64 (16.40)	13.85 (13.65)	12.98 (12.80)	367.07	351.90	263.58	232.32
	P95	120.30	24.08 (20.02)	22.81 (18.96)	17.59 (14.62)	15.80 (13.14)	680.31	619.95	377.94	334.97
Vegetables (g)	Mean	221.59	−0.17 (−0.08)	0 (0)	−0.07 (−0.03)	0.13 (0.06)	45.31	38.12	36.83	28.34
	P05	92.77	−44.38 (47.84)	−42.73 (46.07)	−33.69 (36.32)	−32.67 (35.22)	1990.09	1853.40	1160.65	1096.74
	P10	115.60	−42.02 (36.35)	−41.91 (36.25)	−33.77 (29.21)	−32.45 (28.07)	1785.84	1783.45	1165.28	1072.67
	P25	152.07	−31.18 (20.50)	−29.18 (19.19)	−25.29 (16.63)	−22.54 (14.82)	998.71	872.37	653.75	531.96
	P50	205.82	−11.89 (5.78)	−9.93 (5.08)	−8.79 (4.96)	−6.71 (3.66)	184.77	143.52	136.72	83.84
	P75	276.24	16.40 (6.23)	15.40 (5.60)	12.80 (4.95)	12.34 (4.47)	419.00	308.78	283.12	208.32
	P90	342.43	61.85 (18.06)	58.61 (17.12)	49.45 (14.44)	44.5 (13.00)	4146.79	3697.01	2717.41	2059.91
	P95	394.86	88.21 (22.34)	87.18 (22.08)	72.94 (18.47)	67.12 (17.00)	8194.88	8082.75	5882.91	4812.53

MSE—Mean square error; C2—Two consecutive 24 h recalls; NC2—Two non-consecutive 24 h recalls; C3—Three consecutive 24 h recalls; NC3—Three non-consecutive 24 h recalls.

**Table 3:** Equivalence testing for mean and percentiles of the estimates via scenarios NC2 and C3.

Dietary Components	90% Confidence Interval (%)							
	Mean	P5	P10	P25	P50	P75	P90	P95
Energy	(−0.73, 0.77) *	(−4.19, −2.50) **	(−2.60, −1.40) **	(−1.44, −0.10) **	(−0.94, 0.64) *	(−0.14, 1.76) **	(−0.21, 1.88) **	(0.40, 2.40) **
Fat	(−1.00, −0.01) *	(−12.70, −9.96) ***	(−9.59, −7.59) ***	(−7.61, −6.01) ***	(−3.31, −2.05) **	(−0.53, 0.86) *	(2.58, 4.39) **	(1.95, 3.98) **
Protein	(−0.55, 0.73) *	(−3.87, −2.29) **	(−2.39, −1.15) **	(−2.01, −0.98) **	(−0.50, 0.64) *	(−0.60, 0.82) *	(0.00, 2.05) **	(−0.32, 2.25) **
CHO	(−0.77, 1.13) **	(−4.00, −2.53) **	(−3.26, −2.10) **	(−1.43, 0.26) **	(−0.58, 1.53) **	(−0.34, 1.98) **	(−0.23, 2.01) **	(−0.70, 2.02) **
Fiber	(−0.64, 0.66) *	(−8.25, −6.99) ***	(−5.13, −4.13) **	(−4.32, −3.26) **	(−1.99, −0.49) **	(−0.05, 1.58) **	(0.97, 2.71) **	(2.64, 4.56) **
Cholesterol	(−1.43, 0.49) **	(−68.21, −54.79)	(−44.4, −36.58)	(−10.10, −6.30) ***	(−2.24, −0.07) **	(−0.01, 1.71) **	(1.81, 3.70) **	(3.62, 5.91) **
Calcium	(−0.50, 0.22) *	(−7.14, −4.58) **	(−6.28, −4.16) **	(−4.08, −2.78) **	(−1.78, −0.74) **	(−0.47, 0.53) *	(1.44, 3.21) **	(0.78, 3.22) **
Iron	(−1.34, 1.6) **	(−4.14, −2.73) **	(−2.84, −1.30) **	(−2.80, −1.10) **	(−2.58, −0.26) **	(−1.70, 1.61) **	(−1.02, 3.28) **	(1.36, 6.83) **
Zinc	(−0.65, 1.00) *	(−3.48, −1.56) **	(−2.28, −0.58) **	(−1.95, −0.29) **	(−1.49, 0.08) **	(−0.31, 1.44) **	(−0.44, 1.61) **	(0.18, 2.59) **
Magnesium	(−0.66, 0.88) *	(−4.64, −2.89) **	(−3.03, −1.70) **	(−2.13, −0.87) **	(−1.35, 0.16) **	(−0.63, 1.24) **	(0.59, 2.74) **	(0.71, 3.18) **
Sodium	(−0.87, 0.38) *	(−12.03, −7.48) ***	(−10.02, −7.13) ***	(−7.78, −6.23) ***	(−5.86, −4.55) **	(−0.89, 0.67) *	(2.41, 4.68) **	(3.43, 6.36) **
Potassium	(−0.82, 0.84) *	(−3.88, −2.35) **	(−2.30, −1.16) **	(−1.58, −0.23) **	(−0.71, 0.88) *	(−1.07, 0.60) **	(−0.30, 1.94) **	(0.45, 3.38) **
Phosphorus	(−0.56, 0.80) *	(−3.61, −2.27) **	(−2.45, −1.23) **	(−1.20, 0.10) **	(−0.44, 0.79) *	(−0.13, 1.33) **	(−0.36, 1.78) **	(−0.26, 1.82) **
Vitamin A	(−2.43, 1.95) **	(−17.22, −13.56)	(−13.69, −10.36)	(−8.56, −5.42) ***	(−5.65, −1.90) **	(−4.07, 1.03) **	(−0.72, 5.87) ***	(1.05, 7.36) **
Vitamin C	(−1.16, 1.81) **	(−16.40, −11.35)	(−10.16, −5.99) ***	(−6.67, −2.91) **	(−3.05, 0.17) **	(−0.07, 2.81) **	(0.58, 4.25) **	(2.08, 5.49) **
Vitamin E	(−1.55, 0.89) **	(−7.62, −5.56) ***	(−6.51, −4.58) **	(−6.09, −3.73) **	(−4.32, −1.65) **	(−2.07, 0.69) **	(1.18, 4.43) **	(4.69, 8.18) **
Vitamin B1	(−0.48, 0.81) *	(−8.87, −6.87) ***	(−6.05, −4.60) **	(−3.06, −1.46) **	(−1.08, 0.63) **	(0.03, 1.69) **	(1.65, 3.46) **	(0.99, 2.83) **
Vitamin B2	(−0.65, 0.51) *	(−3.70, −1.91) **	(−4.87, −3.39) **	(−2.35, −0.87) **	(−1.25, 0.13) **	(−0.17, 1.01) **	(−0.1, 1.53) **	(0.51, 2.31) **
Vitamin B3	(−0.60, 0.67) *	(−3.80, −2.13) **	(−3.35, −1.92) **	(−2.58, −1.36) **	(−0.88, 0.20) *	(−0.60, 0.93) *	(−0.4, 1.57) **	(0.63, 3.51) **
Vitamin B9	(−1.24, 0.53) **	(−11.09, −8.39) ***	(−8.25, −6.41) ***	(−4.97, −3.01) **	(−2.57, −0.45) **	(−0.20, 1.78) **	(0.44, 2.47) **	(1.99, 4.48) **
Wheat	(−0.54, 1.50) **	(−99.85, −91.18)	(−25.27, −20.14)	(−6.03, −3.12) **	(0.08, 2.27) **	(0.74, 3.55) **	(1.27, 3.80) **	(0.61, 3.24) **
Pork	(−1.99, 0.07) **	-	-	(−102.83, −96.17)	(−15.3, −10.15)	(0.43, 2.53) **	(2.59, 5.19) **	(7.54, 10.49) ***
Vegetables	(−0.65, 0.72) *	(−17.45, −13.16)	(−11.50, −8.40) ***	(−3.92, −2.21) **	(−1.45, 0.29) **	(0.12, 1.67) **	(1.32, 3.36) **	(1.87, 4.22) **
Milk	(−2.92, 2.39) **	-	-	-	(−107.33, −92.67)	(−6.01, −0.11) ***	(0.75, 5.19) ***	(−1.46, −0.49) **
Beans	(−2.33, 1.94) **	-	(−114.93, −85.07)	(−30.10, −23.40)	(−9.45, −5.95) ***	(−4.97, −0.54) **	(0.96, 6.46) ***	(8.39, 13.93) ***

CHO—Carbohydrate; C3—Three consecutive 24 h recalls; NC2—Two non-consecutive 24 h recalls; *—Statistically significant by equivalence testing (*p* < 0.05) when equivalence testing with equivalence margins of 1% of scenario C3 estimates (for all paraments); **—Statistically significant by equivalence testing (*p* < 0.05) when equivalence testing with equivalence margins of 5% of scenario C3 estimates (for all paraments); ***—Statistically significant by equivalence testing (*p* < 0.05) when equivalence testing with equivalence margins of 10% of scenario C3 estimates (for all paraments).

## Data Availability

The data presented in this study are non-public.

## Supplementary Materials

The following supporting information can be downloaded at: https://www.mdpi.com/article/10.3390/nu14091960/s1, Figure S1: Boxplot of biases of intake calculated for all dietary components based on each scenario with WPM method; Figure S2: Mean relative bias of the percentiles (from 1st to 99th) of intake calculated for all dietary components based on each scenario with WPM method; Figure S3: The mean relative bias of the percentiles (from 1st to 99th) of intake calculated for all dietary components based on each scenario with WPM and NCI method; Table S1: The mean bias, mean relative bias, and MSE of estimates obtained with each scenario. All selected dietary components were included in supplementary tables and figures.
