# Supplementary material for: Comparison of the 24 h Dietary Recall of Two Consecutive Days, Two Non-Consecutive Days, Three Consecutive Days, and Three Non-Consecutive Days for Estimating Dietary Intake of Chinese Adult
Source: Nutrients. 2022 May 7;14(9):1960. doi: 10.3390/nu14091960 (PMC9103339; doi:10.3390/nu14091960)
Supplement: Supplementary file 1 [file nutrients-14-01960-s001.zip › FigureS1.pdf]

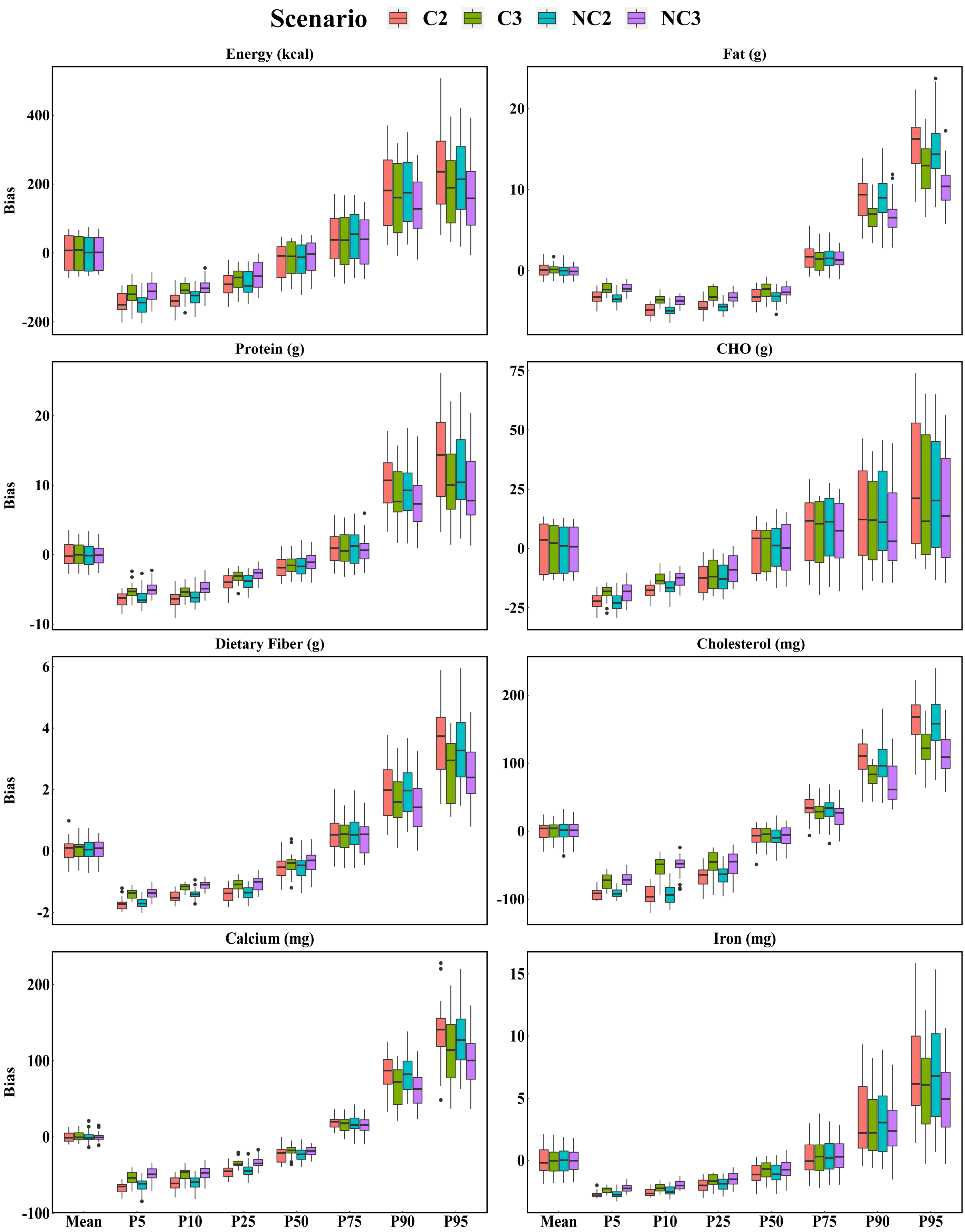

Figure S1. Boxplot of biases of intake calculated for all dietary components based on each scenario with WPM method. CHO—Carbohydrate.

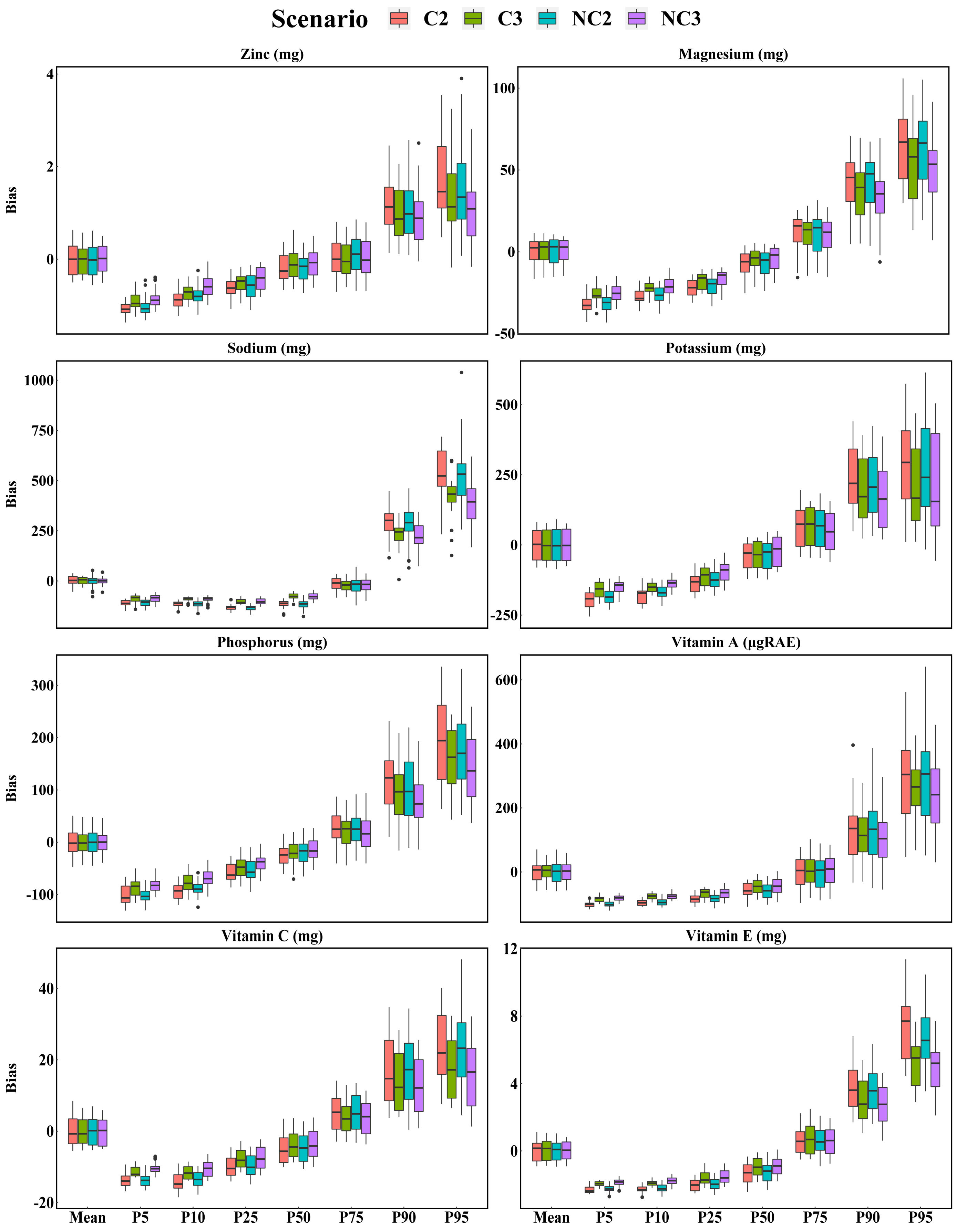

Figure S1. *Cont.*

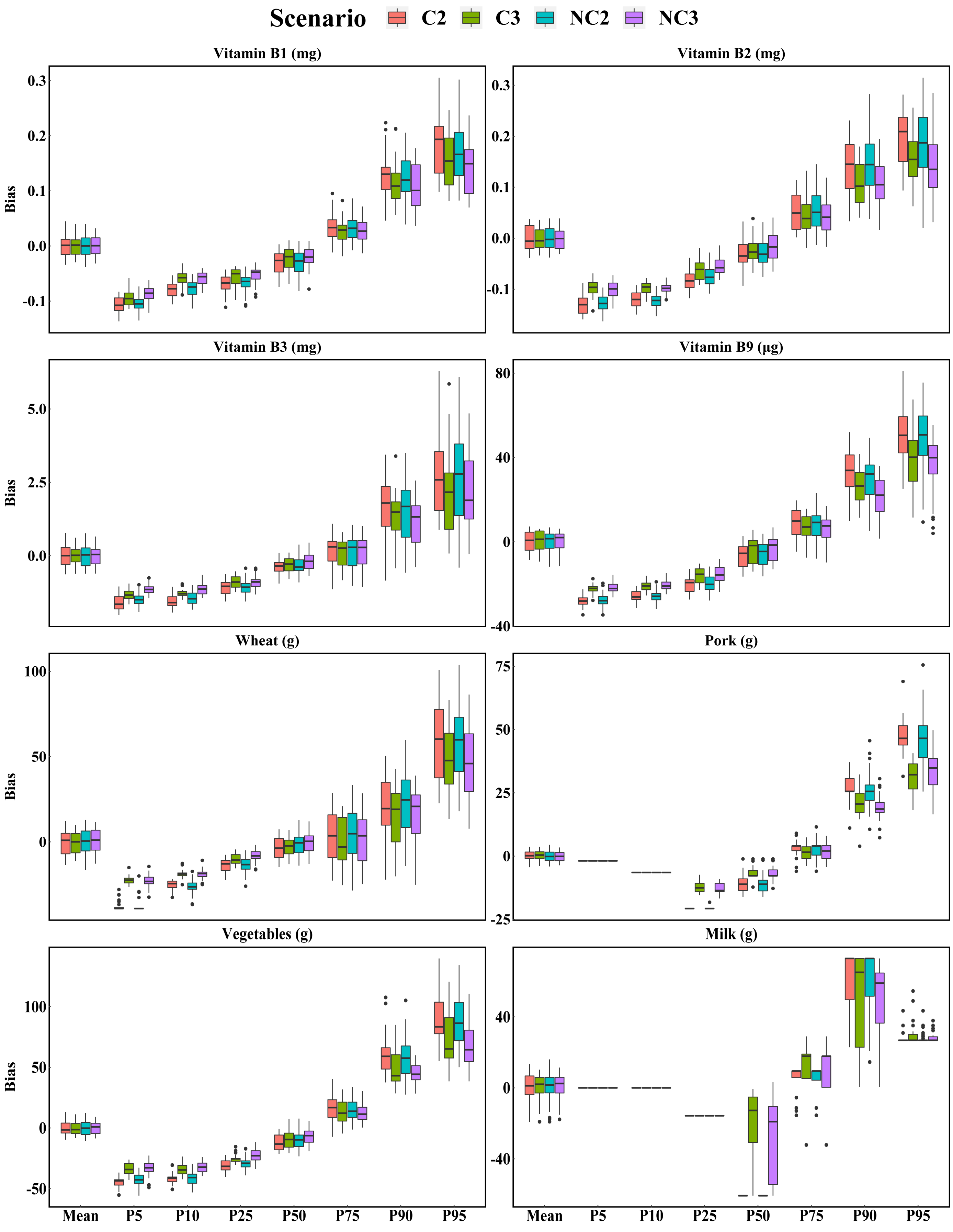

Figure S1. *Cont.*

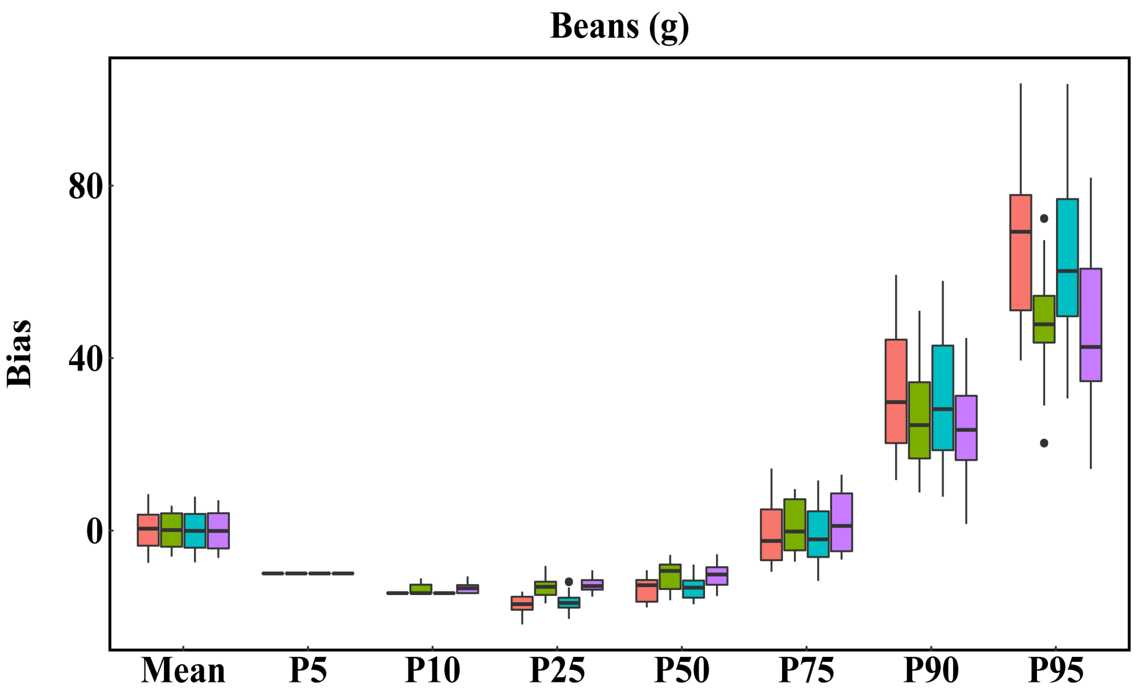

Figure S1. *Cont.*
