# Supplementary material for: Comparison of the 24 h Dietary Recall of Two Consecutive Days, Two Non-Consecutive Days, Three Consecutive Days, and Three Non-Consecutive Days for Estimating Dietary Intake of Chinese Adult
Source: Nutrients. 2022 May 7;14(9):1960. doi: 10.3390/nu14091960 (PMC9103339; doi:10.3390/nu14091960)
Supplement: Supplementary file 1 [file nutrients-14-01960-s001.zip › FigureS3.pdf]

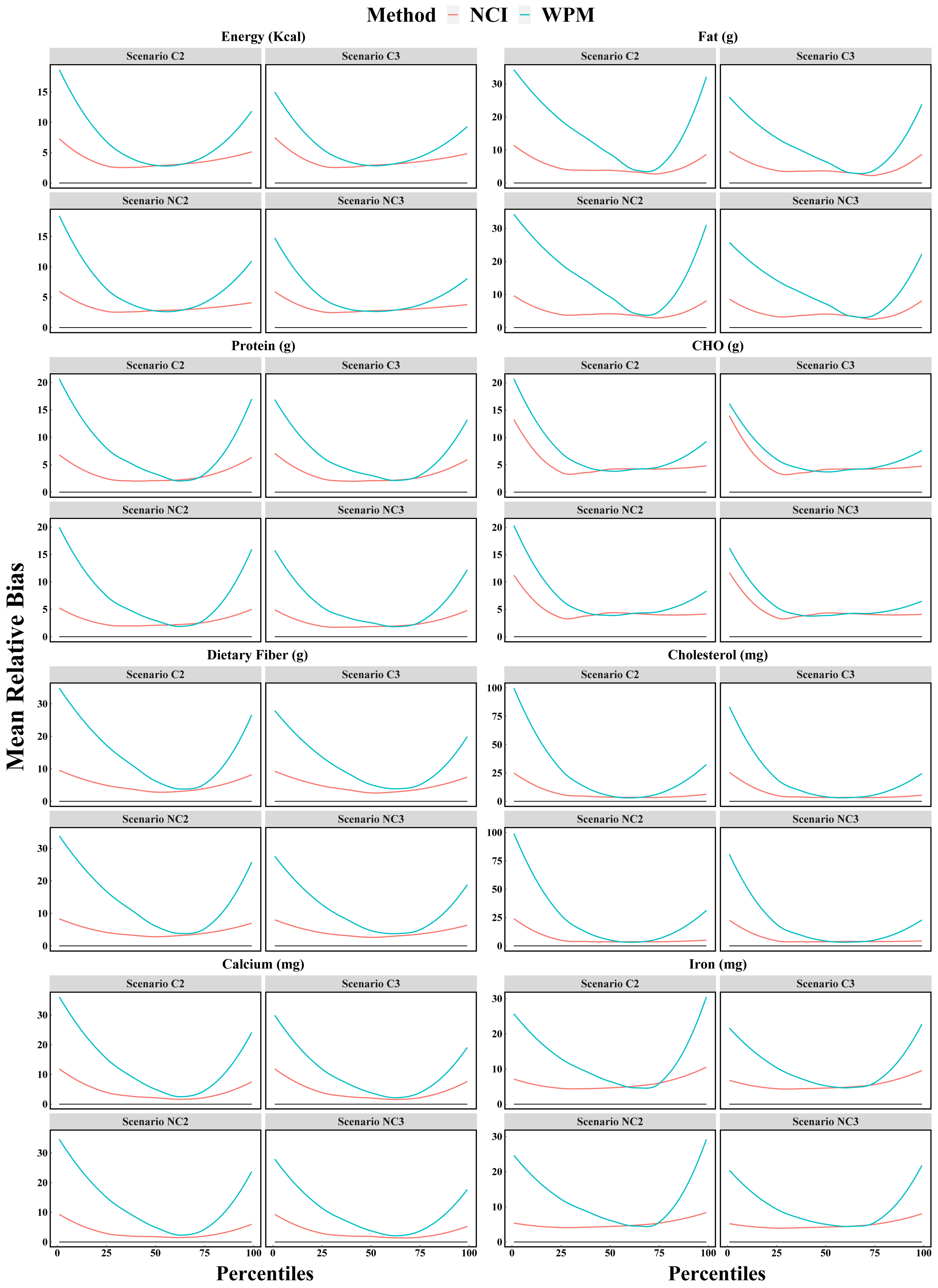

Figure S3. The mean relative bias of the percentiles (from 1st to 99th) of intake calculated for all dietary components based on each scenario with WPM and NCI method.

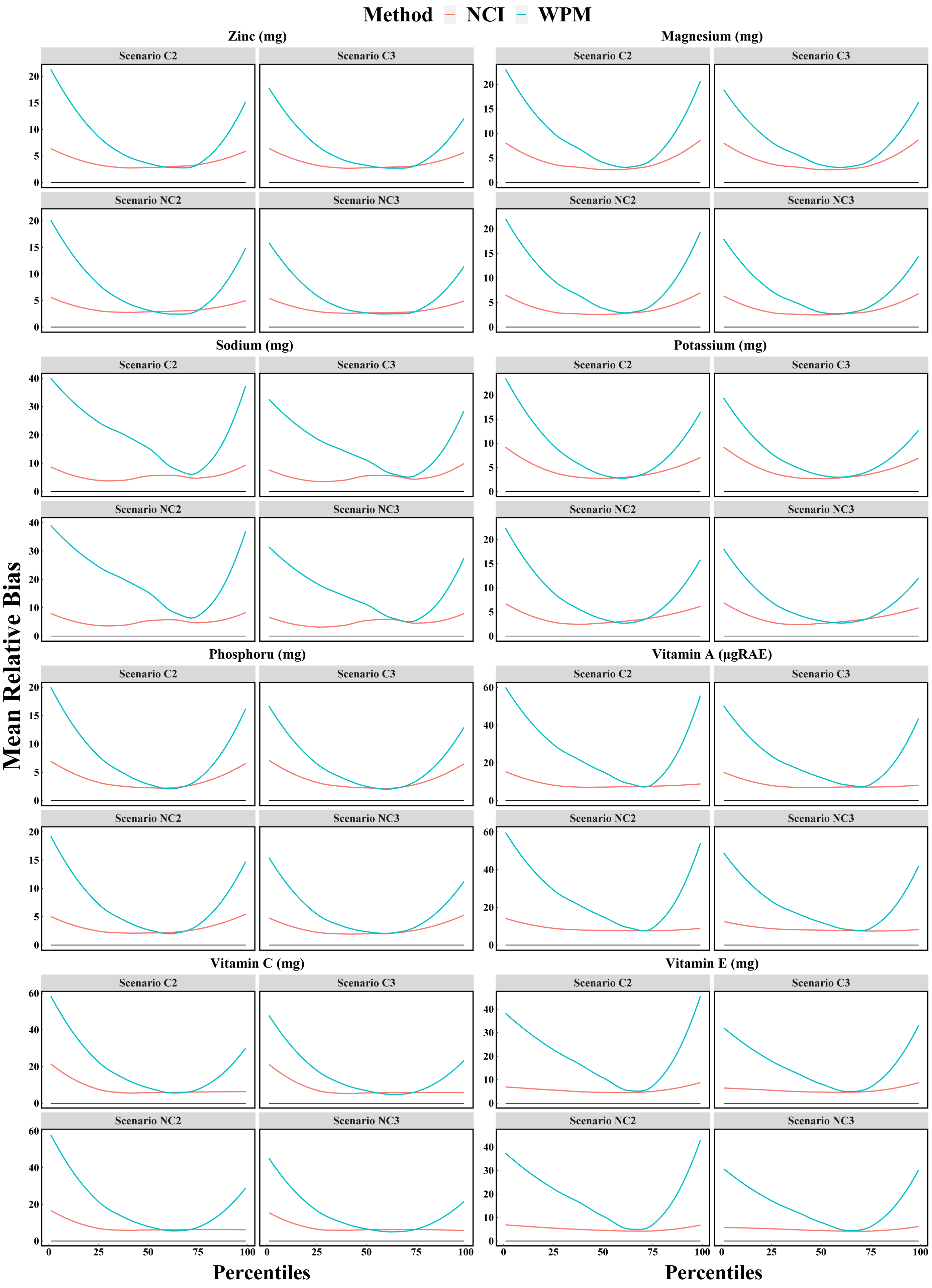

Figure S3. *Cont.*

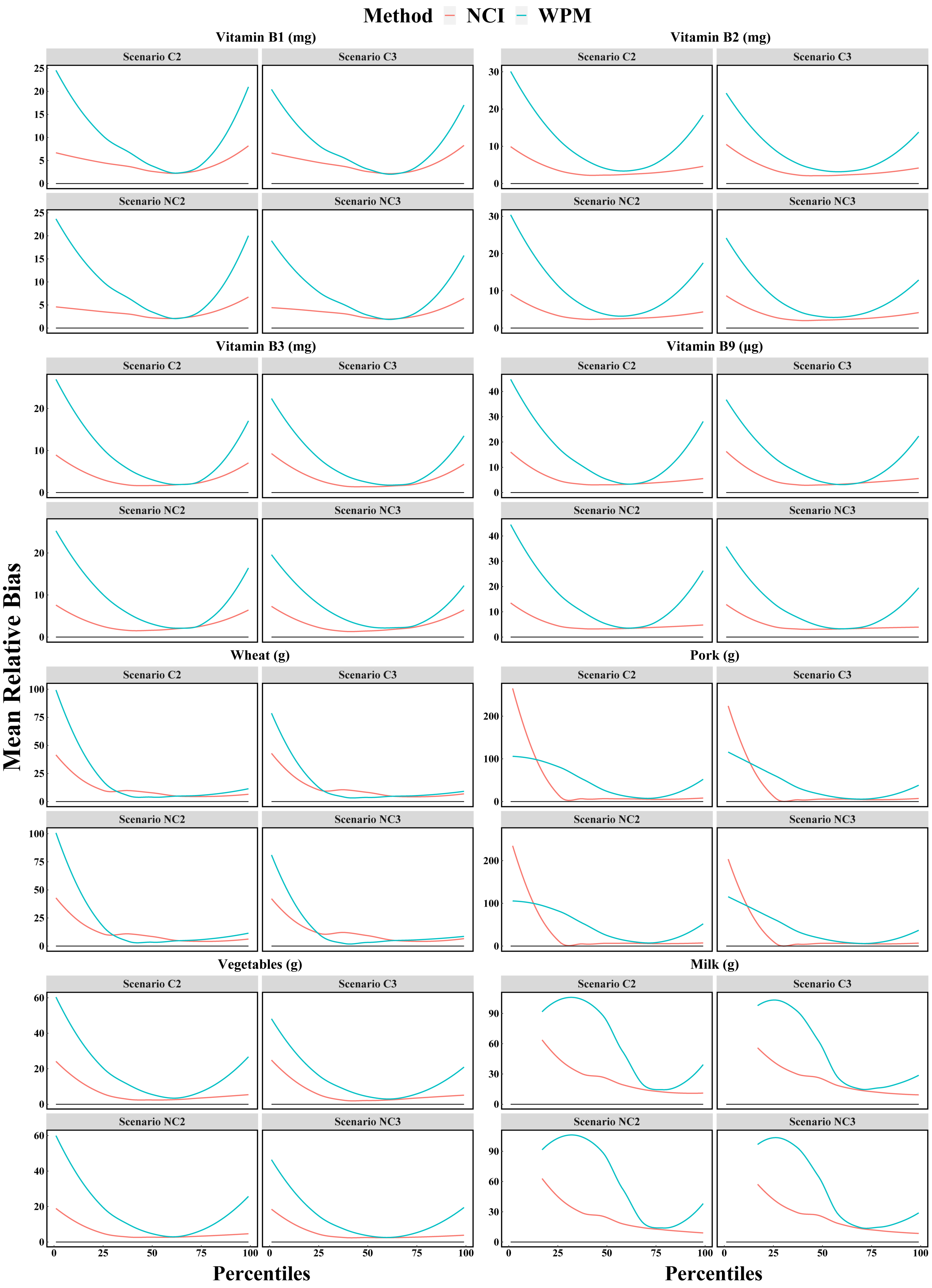

Figure S3. *Cont.*

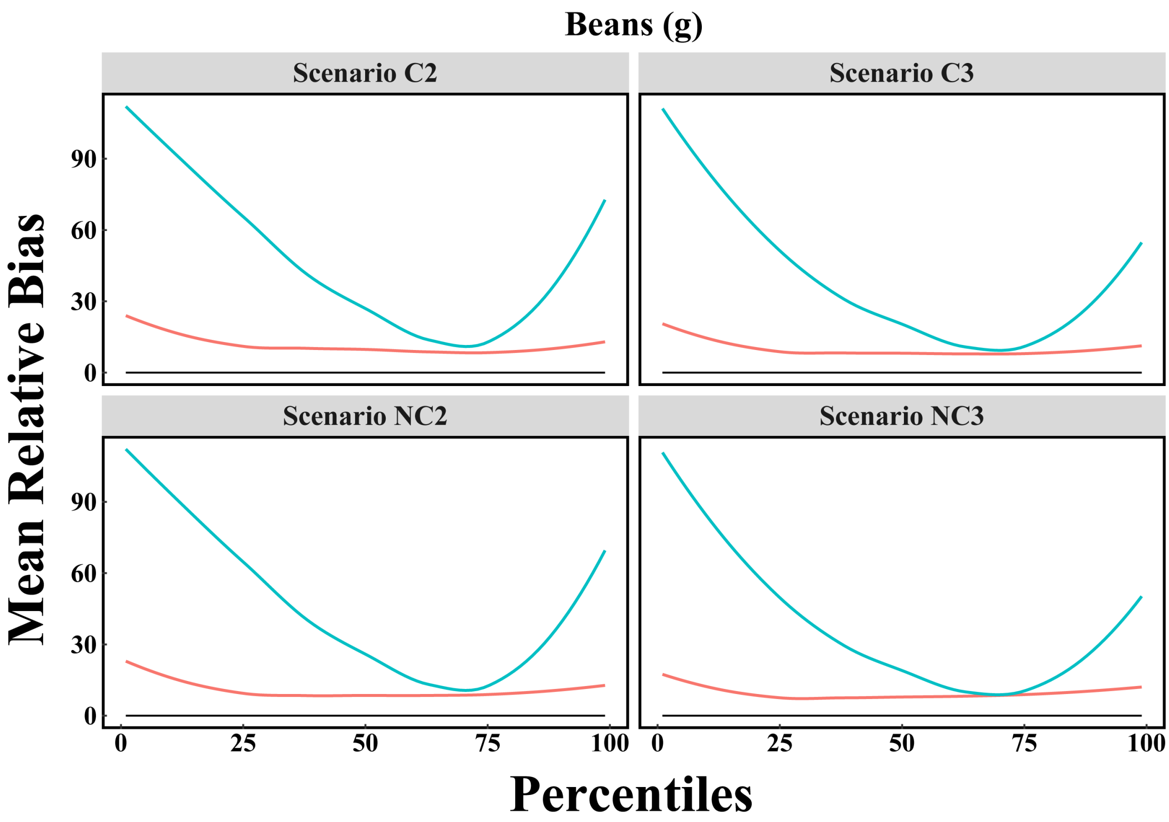

Figure S3. *Cont.*
