# Supplementary material for: Comparison of the 24 h Dietary Recall of Two Consecutive Days, Two Non-Consecutive Days, Three Consecutive Days, and Three Non-Consecutive Days for Estimating Dietary Intake of Chinese Adult
Source: Nutrients. 2022 May 7;14(9):1960. doi: 10.3390/nu14091960 (PMC9103339; doi:10.3390/nu14091960)
Supplement: Supplementary file 1 [file nutrients-14-01960-s001.zip › Table S1.pdf]

Tabel S1 The mean bias, mean relative bias and MSE of estimates obtained with each scenario.

Dietary components	Parameter	True Value	Mean Bias (Mean Relative Bias %)				MSE			
			C2	NC2	C3	NC3	C2	NC2	C3	NC3
Energy(kcal)	Mean	1591.74	0.19(0.01)	0.56(0.03)	0.23(0.01)	0.70(0.04)	2548.70	2303.38	2530.30	2253.84
	P5	981.83	-145.27(14.80)	-146.29(14.90)	-117.41(11.96)	-113.96(11.61)	22085.48	22173.01	15012.37	13765.79
	P10	1091.76	-137.60(12.60)	-128.79(11.80)	-109.11(9.99)	-103.07(9.44)	19834.24	17134.44	12577.09	11240.45
	P25	1286.44	-92.05(7.16)	-86.15(6.70)	-76.87(5.98)	-65.31(5.08)	9598.23	8496.23	7023.83	5639.67
	P50	1543.51	-25.92(2.77)	-20.59(2.79)	-18.35(3.05)	-14.65(2.70)	2831.76	2761.74	2957.89	2416.41
	P75	1858.45	46.62(3.84)	49.09(3.88)	33.68(3.99)	33.98(3.52)	7588.07	7518.43	7232.85	5923.83
	P90	2147.91	187.06(8.71)	173.00(8.05)	153.73(7.20)	137.99(6.48)	47445.66	38989.42	34995.62	26421.90
	P95	2388.49	239.31(10.02)	221.78(9.29)	185.75(7.78)	163.86(6.87)	69300.09	60093.31	45953.04	36221.36
Fat(g)	Mean	37.54	0.10(0.26)	-0.03(-0.09)	0.16(0.42)	-0.07(-0.19)	0.70	0.59	0.53	0.40
	P5	13.72	-3.29(23.97)	-3.45(25.14)	-2.14(15.57)	-2.17(15.83)	11.44	12.27	5.11	5.06
	P10	18.51	-4.91(26.54)	-4.90(26.48)	-3.62(19.57)	-3.77(20.38)	24.79	24.37	13.54	14.58
	P25	25.21	-4.41(17.49)	-4.53(17.99)	-3.02(11.99)	-3.25(12.90)	20.16	21.00	9.91	11.03
	P50	35.72	-3.2(8.95)	-3.35(9.37)	-2.46(6.88)	-2.61(7.30)	11.25	11.82	7.09	7.27
	P75	47.41	1.68(3.95)	1.58(3.58)	1.50(3.46)	1.34(3.19)	5.41	4.33	4.39	3.12
	P90	59.45	9.01(15.15)	9.05(15.23)	6.75(11.35)	6.62(11.13)	86.92	88.72	48.87	48.05
	P95	67.16	15.74(23.43)	14.94(22.25)	12.58(18.73)	10.43(15.53)	259.24	234.01	169.23	115.35
Protein(g)	Mean	66.47	-0.01(-0.01)	0.02(0.03)	-0.04(-0.06)	0.03(0.05)	3.34	2.97	3.16	2.77
	P5	38.46	-6.48(16.84)	-6.31(16.40)	-5.29(13.75)	-4.93(12.82)	43.03	40.86	29.42	25.33
	P10	43.48	-6.42(14.76)	-6.04(13.88)	-5.36(12.33)	-4.64(10.67)	42.73	37.34	29.73	22.72
	P25	51.70	-3.98(7.69)	-3.84(7.42)	-3.11(6.01)	-2.72(5.27)	17.24	15.82	10.54	8.30
	P50	63.66	-1.78(3.18)	-1.48(2.78)	-1.53(2.75)	-0.93(2.34)	5.56	4.33	4.28	3.01
	P75	79.31	1.24(2.68)	1.06(2.68)	0.98(2.56)	0.69(2.14)	7.15	6.49	6.44	4.58
	P90	91.21	10.33(11.32)	9.48(10.39)	8.46(9.28)	7.71(8.45)	124.67	107.60	87.89	75.06
	P95	103.04	13.92(13.51)	11.79(11.44)	10.69(10.38)	9.26(8.99)	237.60	175.03	147.45	113.17
CHO(g)	Mean	247.87	-0.17(-0.07)	0.19(0.08)	-0.26(-0.10)	0.31(0.12)	97.51	90.99	96.08	89.11
	P5	136.92	-22.35(16.33)	-22.63(16.53)	-18.77(13.71)	-18.13(13.24)	514.20	524.70	363.85	345.42
	P10	150.43	-17.98(11.95)	-16.57(11.02)	-12.89(8.57)	-12.93(8.60)	331.97	285.81	174.98	178.11
	P25	182.75	-12.77(6.99)	-11.97(6.55)	-10.97(6)	-8.74(4.85)	203.7	177.34	158.66	111.74
	P50	226.53	-0.39(3.98)	0.39(3.88)	-0.68(3.87)	0.75(4.11)	92.54	97.25	85.09	100.30
	P75	297.98	7.29(4.77)	8.61(4.86)	6.11(4.84)	7.05(4.40)	261.60	274.90	254.93	229.70
	P90	377.20	15.08(5.07)	15.14(4.69)	11.67(4.77)	10.26(4.1)	595.71	534.69	475.94	405.80
	P95	428.02	27.14(6.50)	24.46(6.20)	21.50(5.79)	16.79(5.32)	1438.60	1199.31	1139.48	808.03
Dietary Fiber(g)	Mean	10.42	0.01(0.07)	0(0.02)	0(0.01)	0(-0.02)	0.16	0.16	0.14	0.15
	P5	5.46	-1.73(31.63)	-1.70(31.11)	-1.39(25.42)	-1.37(25.01)	3.03	2.91	1.95	1.89
	P10	6.04	-1.49(24.71)	-1.42(23.42)	-1.19(19.71)	-1.11(18.4)	2.26	2.03	1.43	1.26
	P25	7.81	-1.39(17.82)	-1.36(17.43)	-1.11(14.18)	-1.06(13.54)	1.99	1.9	1.27	1.17
	P50	9.79	-0.57(6.08)	-0.55(5.83)	-0.43(5.07)	-0.40(4.5)	0.47	0.47	0.33	0.32
	P75	12.38	0.53(5.31)	0.56(5.45)	0.46(5.16)	0.49(5.36)	0.68	0.69	0.54	0.60
	P90	15.52	1.99(12.84)	1.97(12.70)	1.66(10.66)	1.45(9.35)	4.75	4.61	3.55	2.90
	P95	17.50	3.52(20.10)	3.36(19.17)	2.63(15.03)	2.55(14.57)	13.72	12.62	7.82	7.34
Cholesterol(mg)	Mean	370.29	0.80(0.22)	-0.45(-0.12)	1.28(0.35)	-0.72(-0.19)	192.65	232.95	151.17	228.50
	P5	102.38	-92.05(89.91)	-91.17(89.06)	-73.27(71.57)	-70.68(69.04)	8533.76	8353.78	5498.00	5100.34
	P10	149.13	-94.46(63.34)	-92.85(62.26)	-54.56(36.58)	-50.06(33.57)	9152.71	8811.25	3313.17	2678.14
	P25	254.40	-67.6(26.57)	-65.51(25.75)	-48.63(19.12)	-49.89(19.61)	4822.29	4504.73	2740.99	2891.46
	P50	353.17	-8.74(3.95)	-10.39(4.13)	-6.40(3.38)	-8.07(3.81)	334.40	358.69	232.61	324.21
	P75	473.24	35.74(7.67)	31.76(6.90)	27.50(5.89)	23.47(5.47)	1608.28	1326.65	1025.86	872.72
	P90	593.55	106.65(17.97)	100.01(16.85)	81.43(13.72)	69.36(11.69)	12057.88	10794.09	6972.83	5549.41
	P95	672.92	164.16(24.39)	159.7(23.73)	121.84(18.11)	113.63(16.89)	28138.34	27029.16	15729.61	13690.12
Calcium(mg)	Mean	427.04	0.34(0.08)	-0.10(-0.02)	0.50(0.12)	-0.23(-0.05)	45.75	39.59	38.00	29.36
	P5	218.74	-66.73(30.51)	-63.21(28.9)	-53.53(24.47)	-49.26(22.52)	4500.62	4070.07	2938.8	2510.05
	P10	249.64	-61.60(24.68)	-59.78(23.95)	-49.32(19.76)	-46.59(18.66)	3878.33	3646.45	2514.09	2252.17
	P25	309.89	-45.42(14.66)	-44.14(14.24)	-34.70(11.20)	-33.59(10.84)	2127.21	2009.32	1234.90	1176.94
	P50	406.26	-23.39(5.76)	-23.47(5.78)	-18.60(4.58)	-18.45(4.54)	646.62	620.23	407.31	380.45
	P75	515.45	19.55(3.79)	16.86(3.41)	16.70(3.30)	15.12(3.18)	465.04	410.34	376.32	342.73
	P90	631.10	84.5(13.39)	82.97(13.15)	66.74(10.58)	61.71(9.78)	7735.87	7471.48	5222.41	4205.99
	P95	720.27	135.74(18.85)	130.36(18.10)	113.66(15.78)	101.3(14.06)	20266.30	18634.97	14876.69	11559.24

Tabe Sl. *Cont.*

Dietary components	Parameter	True Value	Bias (Relative Bias %)				MSE			
			C2	NC2	C3	NC3	C2	NC2	C3	NC3
Irom (mg)	Mean	19.62	-0.01(-0.04)	0.01(0.05)	-0.02(-0.08)	0.02(0.09)	1.49	1.36	1.43	1.31
	P5	12.00	-2.75(22.96)	-2.72(22.64)	-2.39(19.89)	-2.23(18.55)	7.64	7.46	5.75	5.03
	P10	13.20	-2.57(19.48)	-2.44(18.46)	-2.21(16.73)	-1.97(14.93)	6.71	6.06	4.96	3.99
	P25	15.58	-2.01(12.93)	-1.9(12.19)	-1.63(10.44)	-1.51(9.68)	4.31	3.85	2.85	2.54
	P50	18.81	-1.12(6.11)	-1.05(6.01)	-0.80(4.92)	-0.71(5.11)	1.86	1.85	1.30	1.32
	P75	22.91	0.32(5.63)	0.34(5.44)	0.35(6.14)	0.39(5.34)	2.41	2.39	3.11	2.39
	P90	27.30	3.34(12.53)	3.18(11.98)	2.84(11.07)	2.58(10.95)	20.20	16.99	15.29	13.43
	P95	29.48	7.16(24.29)	6.99(23.71)	5.55(18.94)	5.04(17.14)	67.81	65.22	42.77	36.17
Zinc (mg)	Mean	9.90	-0.01(-0.07)	0(0.04)	-0.01(-0.14)	0.01(0.07)	0.12	0.11	0.11	0.10
	P5	5.63	-1.08(19.13)	-1.03(18.30)	-0.91(16.19)	-0.85(15.08)	1.18	1.09	0.87	0.76
	P10	6.17	-0.87(14.11)	-0.8(12.89)	-0.72(11.63)	-0.58(9.33)	0.79	0.67	0.54	0.39
	P25	7.53	-0.63(8.42)	-0.58(7.71)	-0.50(6.66)	-0.42(5.63)	0.44	0.40	0.29	0.24
	P50	9.31	-0.19(3.36)	-0.19(2.87)	-0.12(3.15)	-0.09(2.62)	0.13	0.11	0.13	0.09
	P75	11.82	0.04(2.98)	0.08(3.06)	0.02(2.93)	0.03(2.82)	0.17	0.19	0.17	0.17
	P90	14.06	1.16(8.28)	1.07(7.61)	0.98(6.99)	0.87(6.21)	1.86	1.54	1.36	1.08
	P95	16.06	1.75(10.89)	1.52(9.48)	1.28(8.1)	1.13(7.09)	3.90	3.03	2.40	1.87
Magnesium (mg)	Mean	279.06	-0.06(-0.02)	0.13(0.05)	-0.17(-0.06)	0.14(0.05)	81.16	75.95	79.44	74.88
	P5	172.49	-32.3(18.73)	-31.85(18.46)	-26.34(15.27)	-25.14(14.58)	1067.43	1040.58	723.69	655.52
	P10	189.59	-27.63(14.57)	-26.15(13.79)	-22.19(11.71)	-20.97(11.06)	786.96	706.20	508.28	469.28
	P25	225.24	-21.89(9.72)	-20.91(9.28)	-17.80(7.9)	-16.51(7.33)	507.35	467.72	339.53	304.49
	P50	270.03	-8.13(3.23)	-7.36(2.96)	-5.8(2.59)	-4.42(2.3)	142.45	120.17	99.61	76.21
	P75	324.03	11.46(4.83)	10.48(4.51)	9.46(4.94)	9.00(4.30)	290.68	260.38	283.12	226.18
	P90	376.32	41.09(10.92)	41.81(11.11)	34.96(9.29)	31.82(8.57)	2085.69	2061.35	1589.29	1345.59
	P95	409.26	65.99(16.12)	62.99(15.39)	53.99(13.19)	48.94(11.96)	4824.27	4513.57	3460.27	2858.36
Sodium (mg)	Mean	901.42	1.34(0.15)	-0.12(-0.01)	2.06(0.23)	-0.48(-0.05)	631.87	597.59	336.23	327.51
	P5	301.27	-112.25(37.26)	-109.76(36.43)	-89.05(29.56)	-87.53(29.05)	12958.65	12402.90	8413.25	8066.79
	P10	371.51	-116.79(31.44)	-115.78(31.16)	-91.79(24.71)	-91.56(24.65)	13919.95	13714.87	8585.24	8590.26
	P25	537.73	-132.34(24.61)	-133.69(24.86)	-103.26(19.20)	-103.18(19.19)	17719.29	18067.40	10834.99	10861.11
	P50	808.07	-115.44(14.29)	-115.17(14.25)	-77.14(9.55)	-77.51(9.59)	13744.83	13671.79	6247.53	6231.88
	P75	1190.64	-15.84(2.55)	-22.2(2.96)	-20.92(2.59)	-20.76(2.57)	1452.01	2013.14	1407.19	1520.81
	P90	1531.49	289.40(18.90)	286.13(18.68)	223.89(14.62)	222.14(14.51)	90381.98	89051.91	54808.45	54519.73
	P95	1784.09	535.93(30.04)	523.17(29.32)	415.60(23.29)	393.43(22.05)	304828.20	292408.40	185574.70	167047.70
Potassium (mg)	Mean	1656.70	0.07(0)	0.10(0.01)	-0.10(-0.01)	0.30(0.02)	3258.73	3144.80	3203.10	3052.69
	P5	998.88	-193.84(19.41)	-185.65(18.59)	-159.5(15.97)	-148.94(14.91)	38311.02	35067.40	26364.52	22718.79
	P10	1119.60	-178.89(15.98)	-167.52(14.96)	-150.79(13.47)	-136.89(12.23)	32792.73	28622.93	23064.33	19050.10
	P25	1333.47	-134.78(10.11)	-123.59(9.27)	-112.54(8.44)	-95.51(7.16)	19330.11	16450.42	13670.92	10301.32
	P50	1597.43	-38.32(3.07)	-35.89(3.04)	-37.21(3.15)	-24.14(3.09)	3880.79	3759.59	4127.07	3251.57
	P75	1930.12	68.66(4.24)	62.14(3.76)	66.83(4.24)	50.16(3.66)	10468.08	8405.42	9444.06	7320.60
	P90	2269.66	228.69(10.08)	215.80(9.51)	195.50(8.61)	176.49(7.78)	66645.00	58896.07	51795.01	44803.84
	P95	2557.98	291.99(11.42)	272.25(10.66)	218.98(8.56)	216.54(8.61)	111862.40	102786.20	72060.84	75703.08
Phosphorus (mg)	Mean	976.50	-0.48(-0.05)	0.39(0.04)	-0.80(-0.08)	0.79(0.08)	782.79	722.82	769.02	700.48
	P5	610.11	-102.06(16.73)	-101.87(16.70)	-86.48(14.18)	-82.38(13.5)	10775.16	10546.43	7791.37	6937.30
	P10	674.41	-93.89(13.92)	-88.17(13.07)	-77.19(11.45)	-67.51(10.01)	9062.07	7957.88	6282.96	4827.38
	P25	788.41	-58.47(7.42)	-53.2(6.75)	-49.13(6.23)	-38.49(4.88)	3772.87	3213.81	2812.55	1798.56
	P50	952.28	-25.55(2.95)	-18.76(2.58)	-20.35(2.67)	-14.10(2.44)	1066.90	899.55	952.05	746.62
	P75	1139.44	26.44(3.29)	25.16(3.05)	18.22(2.98)	17.68(2.96)	1940.12	1805.31	1515.16	1704.29
	P90	1313.30	115.21(8.77)	102.68(7.85)	92.65(7.18)	81.78(6.28)	16790.63	14209.80	12594.32	9936.27
	P95	1418.67	194.67(13.72)	173.51(12.23)	161.16(11.36)	143.57(10.12)	44571.04	34753.17	29948.51	24606.41
Vitamin A (μgRAE)	Mean	415.13	0.35(0.09)	-0.5(-0.12)	0.49(0.12)	-0.78(-0.19)	1369.94	1428.52	1219.94	1355.04
	P5	194.64	-101.19(51.99)	-100.47(51.62)	-83.34(42.82)	-81.39(41.82)	10310.13	10156.09	7034.74	6713.83
	P10	224.68	-96.03(42.74)	-94.41(42.02)	-76.61(34.1)	-76.29(33.96)	9299.17	9016.13	5964.67	5906.18
	P25	288.28	-84.37(29.27)	-83.09(28.82)	-67.66(23.47)	-65.78(22.82)	7313.06	7089.38	4816.73	4615.03
	P50	377.66	-58.6(15.52)	-59.09(15.65)	-46.60(12.34)	-45.46(12.07)	3977.19	4119.48	2845.51	2849.18
	P75	502.10	-0.26(8.41)	-2.99(8.86)	4.71(8.59)	1.82(8.79)	2595.59	2781.51	2814.40	2857.93
	P90	650.15	129.24(20.82)	127.87(21.06)	108.33(18.02)	109.57(18.13)	28020.08	27645.74	19369.27	20473.38
	P95	762.83	300.28(39.36)	294.41(38.59)	251.77(33.00)	233.34(30.59)	106654.4	105198.5	75792.43	67715.72

Tabe Sl. *Cont.*

Dietary components	Parameter	True Value	Mean Bias (Mean Relative Bias %)				MSE			
			C2	NC2	C3	NC3	C2	NC2	C3	NC3
Vitamin C (mg)	Mean	65.68	-0.06(-0.09)	0.01(0.01)	-0.21(-0.31)	-0.03(-0.04)	15.88	16.13	14.30	15.65
	P5	28.59	-13.71(47.96)	-13.71(47.97)	-11.32(39.59)	-10.4(36.37)	191.38	191.45	130.65	110.08
	P10	35.41	-14.13(39.91)	-13.44(37.94)	-11.51(32.49)	-10.53(29.75)	205.34	184.91	135.68	115.61
	P25	45.32	-9.88(21.81)	-9.52(21.00)	-7.72(17.03)	-7.39(16.31)	105.04	99.41	66.08	65.57
	P50	61.68	-4.69(8.59)	-4.75(8.07)	-3.92(7.31)	-3.60(6.81)	38.72	36.95	29.25	27.16
	P75	80.23	4.98(6.95)	5.01(6.84)	3.85(5.77)	3.79(5.98)	50.78	50.25	36.61	35.68
	P90	101.46	16.76(16.52)	16.64(16.40)	13.85(13.65)	12.98(12.80)	367.07	351.90	263.58	232.32
	P95	120.30	24.08(20.02)	22.81(18.96)	17.59(14.62)	15.80(13.14)	680.31	619.95	377.94	334.97
Vitamin E (mg)	Mean	11.75	0.03(0.24)	0(-0.04)	0.03(0.29)	-0.01(-0.12)	0.39	0.32	0.39	0.31
	P5	6.47	-2.3(35.58)	-2.25(34.82)	-1.96(30.22)	-1.86(28.71)	5.34	5.12	3.85	3.49
	P10	7.36	-2.27(30.85)	-2.21(29.97)	-1.9(25.85)	-1.77(23.98)	5.20	4.92	3.65	3.17
	P25	9.03	-2.03(22.45)	-1.96(21.73)	-1.6(17.68)	-1.52(16.88)	4.23	3.96	2.71	2.46
	P50	11.3	-1.34(11.83)	-1.26(11.19)	-0.96(8.46)	-0.92(8.21)	2.09	1.91	1.24	1.16
	P75	13.9	0.66(5.99)	0.64(5.87)	0.74(6.66)	0.61(5.83)	1.11	1.06	1.29	0.95
	P90	16.84	3.79(22.52)	3.60(21.4)	3.05(18.09)	2.75(16.34)	16.18	14.72	11.00	8.84
	P95	18.5	7.14(38.61)	6.70(36.21)	5.17(27.97)	4.83(26.10)	54.46	47.87	29.13	25.15
Vitamin B1 (mg)	Mean	0.87	0(-0.09)	0(0.07)	0(-0.09)	0(0.15)	0	0	0	0
	P5	0.50	-0.13(26.27)	-0.13(26.02)	-0.10(19.7)	-0.10(20.34)	0.02	0.02	0.01	0.01
	P10	0.56	-0.12(21.41)	-0.12(21.80)	-0.10(17.40)	-0.10(17.41)	0.01	0.02	0.01	0.01
	P25	0.67	-0.08(12.27)	-0.07(11.2)	-0.06(9.15)	-0.05(8.11)	0.01	0.01	0	0
	P50	0.83	-0.03(4.37)	-0.03(4.06)	-0.02(3.93)	-0.02(3.26)	0	0	0	0
	P75	1.03	0.05(5.02)	0.05(5.37)	0.05(4.59)	0.04(4.36)	0	0	0	0
	P90	1.26	0.14(11.43)	0.15(11.59)	0.11(8.81)	0.11(8.61)	0.02	0.02	0.01	0.01
	P95	1.43	0.20(13.74)	0.18(12.84)	0.15(10.72)	0.14(9.65)	0.04	0.04	0.03	0.02
Vitamin B2 (mg)	Mean	0.81	0(0.02)	0(-0.01)	0(0.06)	0(-0.01)	0	0	0	0
	P5	0.48	-0.11(22.19)	-0.10(21.79)	-0.09(19.53)	-0.09(18.13)	0.01	0.01	0.01	0.01
	P10	0.52	-0.08(15.04)	-0.08(14.83)	-0.06(11.17)	-0.06(11.20)	0.01	0.01	0	0
	P25	0.65	-0.07(10.77)	-0.07(10.51)	-0.06(9.05)	-0.05(8.20)	0.01	0.01	0	0
	P50	0.79	-0.03(3.92)	-0.03(3.76)	-0.02(3.34)	-0.02(2.94)	0	0	0	0
	P75	0.94	0.03(3.79)	0.03(3.48)	0.03(3.17)	0.03(3.08)	0	0	0	0
	P90	1.09	0.13(11.88)	0.13(11.52)	0.12(10.72)	0.11(9.83)	0.02	0.02	0.02	0.01
	P95	1.23	0.18(15.09)	0.17(14.18)	0.15(12.59)	0.14(11.66)	0.04	0.03	0.03	0.02
Vitamin B3 (mg)	Mean	14.04	0(-0.02)	0(0.02)	0(-0.02)	0(0.01)	0.13	0.14	0.1	0.13
	P5	6.86	-1.61(23.52)	-1.49(21.74)	-1.33(19.35)	-1.15(16.83)	2.67	2.26	1.79	1.36
	P10	7.96	-1.55(19.42)	-1.45(18.2)	-1.27(15.99)	-1.13(14.23)	2.45	2.14	1.64	1.32
	P25	10.05	-1.07(10.68)	-1.07(10.66)	-0.89(8.86)	-0.91(9.09)	1.21	1.20	0.84	0.87
	P50	13.00	-0.38(2.96)	-0.35(2.96)	-0.30(2.45)	-0.19(2.16)	0.21	0.20	0.15	0.12
	P75	17.44	0.15(2.79)	0.12(2.88)	0.09(2.51)	0.07(2.89)	0.33	0.33	0.25	0.33
	P90	21.61	1.57(7.7)	1.48(7.03)	1.35(6.45)	1.15(5.56)	3.55	3.08	2.55	2.00
	P95	24.70	2.87(11.61)	2.74(11.15)	2.18(8.85)	2.22(8.99)	10.66	10.14	6.88	6.69
Vitamin B9 (µg)	Mean	144.52	0.24(0.16)	-0.17(-0.12)	0.34(0.24)	-0.30(-0.21)	25.28	27.81	26.53	28.69
	P5	74.92	-28.02(37.40)	-27.60(36.84)	-22.49(30.02)	-21.84(29.16)	793.71	770.79	512.10	484.03
	P10	86.45	-26.03(30.11)	-25.80(29.85)	-21.01(24.30)	-20.54(23.76)	685.68	671.68	447.49	427.45
	P25	108.35	-19.93(18.40)	-19.72(18.20)	-16.03(14.80)	-15.49(14.30)	413.57	402.03	271.64	256.84
	P50	135.34	-6.40(4.90)	-5.71(4.65)	-3.73(3.83)	-3.46(3.84)	67.49	63.70	49.83	45.15
	P75	173.99	9.19(5.62)	8.08(5.53)	6.65(4.79)	5.76(4.70)	134.31	121.28	87.87	84.44
	P90	213.38	33.68(15.78)	29.57(13.86)	26.09(12.23)	21.27(9.97)	1244.39	980.37	755.11	538.83
	P95	243.80	52.21(21.41)	48.91(20.06)	39.74(16.30)	36.37(14.92)	2921.68	2598.00	1794.97	1500.09
Wheat (g)	Mean	170.40	0.31(0.18)	-0.50(-0.29)	0.63(0.37)	53.12	51.35	46.00	45.14	-0.45(-0.27)
	P5	39.09	-38.34(98.08)	-22.33(57.12)	-23.23(59.42)	1414.38	1479.01	505.36	552.21	-37.48(95.88)
	P10	51.68	-26.27(50.83)	-18.8(36.39)	-19.09(36.94)	650.07	703.26	360.92	372.41	-25.31(48.98)
	P25	80.06	-13.28(16.59)	-10.08(12.59)	-8.23(10.28)	209.90	196.07	111.59	79.20	-13.92(17.39)
	P50	142.26	-0.94(3.60)	-2.59(3.63)	-0.78(3.44)	57.74	41.65	40.26	35.71	-3.97(4.56)
	P75	238.70	4.74(5.08)	-0.37(5.22)	1.99(5.22)	217.62	201.33	206.57	196.01	3.13(5.41)
	P90	340.11	21.43(7.17)	12.50(5.95)	15.07(6.23)	662.34	777.97	519.76	538.85	19.00(6.38)
	P95	379.39	57.19(15.08)	48.94(12.90)	46.78(12.33)	4049.47	3835.08	2814.60	2608.27	58.52(15.42)

Tabe Sl. *Cont.*

Dietary components	Parameter	True Value	Bias (Relative Bias %)				MSE			
			C2	NC2	C3	NC3	C2	NC2	C3	NC3
Pork (g)	Mean	48.62	0.16(0.33)	-0.09(-0.18)	0.38(0.78)	-0.10(-0.21)	4.84	4.48	3.66	3.36
	P5	1.86	-1.86(100.00)	-1.86(100.00)	-1.86(100.00)	-1.86(100.00)	3.45	3.45	3.45	3.45
	P10	6.46	-6.46(100.00)	-6.46(100.00)	-6.46(100.00)	-6.46(100.00)	41.74	41.74	41.74	41.74
	P25	20.71	-20.71(100.00)	-20.67(99.8)	-12.41(59.93)	-12.74(61.51)	429.08	427.46	158.87	165.9
	P50	41.11	-10.96(26.67)	-11.09(26.98)	-6.71(16.33)	-6.99(17.01)	135.01	138.29	51.00	54.71
	P75	70.96	2.83(6.03)	2.64(4.81)	1.57(4.03)	1.52(4.34)	22.18	16.57	11.97	13.73
	P90	99.40	26.72(26.88)	25.89(26.05)	21.20(21.33)	19.22(19.34)	748.40	710.94	490.97	397.44
	P95	118.48	47.29(39.92)	45.64(38.52)	32.07(27.07)	34.27(28.93)	2299.31	2179.34	1065.51	1244.73
Vegetables (g)	Mean	221.59	-0.17(-0.08)	0(0)	-0.07(-0.03)	0.13(0.06)	45.31	38.12	36.83	28.34
	P5	92.77	-44.38(47.84)	-42.73(46.07)	-33.69(36.32)	-32.67(35.22)	1990.09	1853.40	1160.65	1096.74
	P10	115.60	-42.02(36.35)	-41.91(36.25)	-33.77(29.21)	-32.45(28.07)	1785.84	1783.45	1165.28	1072.67
	P25	152.07	-31.18(20.50)	-29.18(19.19)	-25.29(16.63)	-22.54(14.82)	998.71	872.37	653.75	531.96
	P50	205.82	-11.89(5.78)	-9.93(5.08)	-8.79(4.96)	-6.71(3.66)	184.77	143.52	136.72	83.84
	P75	276.24	16.40(6.23)	15.40(5.60)	12.80(4.95)	12.34(4.47)	419.00	308.78	283.12	208.32
	P90	342.43	61.85(18.06)	58.61(17.12)	49.45(14.44)	44.5(13.00)	4146.79	3697.01	2717.41	2059.91
	P95	394.86	88.21(22.34)	87.18(22.08)	72.94(18.47)	67.12(17.00)	8194.88	8082.75	5882.91	4812.53
Milk (g)	Mean	77.94	0.09(0.11)	0.04(0.06)	0.25(0.32)	0.09(0.12)	71.63	73.59	64.36	69.34
	P5	0	0(-)	0(-)	0(-)	0(-)	0	0	0	0
	P10	0	0(-)	0(-)	0(-)	0(-)	0	0	0	0
	P25	15.72	-15.72(100.00)	-15.72(100.00)	-15.72(100.00)	-15.72(100.00)	247.19	247.19	247.19	247.19
	P50	60.71	-60.71(100.00)	-60.71(100.00)	-22.5(37.05)	-26.94(44.63)	3686.22	3686.22	1026.48	1191.42
	P75	115.48	3.97(8.93)	3.34(9.48)	7.09(20.06)	5.85(18.98)	111.56	126.05	572.60	528.16
	P90	177.12	61.49(34.72)	60.16(33.97)	53.32(30.10)	47.82(27.00)	4139.85	4012.85	3404.62	2941.05
	P95	223.21	29.04(13.01)	28.31(12.68)	30.79(13.79)	28.02(12.56)	868.04	815.04	1006.70	791.71
Beans (g)	Mean	50.15	0.06(0.12)	0.05(0.11)	0.15(0.30)	0.09(0.18)	19.58	20.09	16.13	19.05
	P5	9.93	-9.93(100.00)	-9.93(100.00)	-9.93(100.00)	-9.93(100.00)	98.61	98.61	98.61	98.61
	P10	14.49	-14.49(100.00)	-14.49(100.00)	-13.51(93.26)	-13.26(91.57)	209.85	209.85	183.92	177.54
	P25	26.86	-17.07(63.56)	-16.79(62.49)	-13.11(48.79)	-12.67(47.16)	295.61	284.85	176.88	163.09
	P50	46.64	-13.59(29.13)	-13.27(28.44)	-10.48(22.48)	-10.36(22.20)	192.07	181.77	119.22	113.72
	P75	67.04	-0.77(8.83)	-0.87(8.30)	1.00(8.60)	1.97(9.41)	44.31	38.51	38.31	48.86
	P90	92.11	32.08(34.82)	30.45(33.06)	26.07(28.30)	24.05(26.11)	1202.82	1118.56	808.87	719.22
	P95	104.32	67.56(64.76)	65.01(62.31)	48.01(46.02)	47.46(45.50)	4916.22	4568.12	2462.65	2564.99
